# Mechanochemical Nitrous Oxide Decomposition

**DOI:** 10.1002/adma.202511666

**Published:** 2025-09-26

**Authors:** Seung‐Hyeon Kim, Li‐Bo Chen, Jae Seong Lee, Ayeon Kim, Jeong‐Min Seo, Jae‐Hoon Baek, Se Jung Lee, Boo‐Jae Jang, Changqing Li, Runnan Guan, Yanhua Shao, Jian Li, Xing‐You Lang, Yung Sam Kim, Gao‐Feng Han, Qing Jiang, Jong‐Beom Baek

**Affiliations:** ^1^ Department of Energy and Chemical Engineering/Center for Dimension‐Controllable Organic Frameworks Ulsan National Institute of Science and Technology (UNIST) Ulsan 44919 Republic of Korea; ^2^ Key Laboratory of Automobile Materials (Jilin University) Ministry of Education Jilin University Changchun 130012 P. R. China; ^3^ Green Circulation R&D Department Korea Institute of Industrial Technology (KITECH) Cheonan 31056 Republic of Korea; ^4^ Department of Chemistry Ulsan National Institute of Science and Technology (UNIST) Ulsan 44919 Republic of Korea

**Keywords:** ball milling, catalyst transformation, greenhouse gas mitigation, mechanochemistry, nitrous oxide decomposition

## Abstract

Nitrous oxide (N_2_O) is one of the top three greenhouse gases, together with carbon dioxide and methane, but it is stable enough not to be easily decomposed under thermocatalytic conditions, even at high temperature (445 °C). Herein, an efficient N_2_O decomposition method is reported using nickel oxide catalyst under mechanochemical conditions operated near ambient temperature. The mechanochemical N_2_O decomposition method exhibited a rapid reaction rate of 1761.3 mL h^−1^ and a high conversion of 99.98% even at 42 °C, compared to the thermochemical method (294.9 mL h^−1^ and 49.16% at 445 °C). Unlike equilibrium thermocatalytic states, the non‐equilibrium mechanocatalytic states induced by intensive dynamic mechanochemical actions are responsible for effective N_2_O decomposition under mild temperature conditions.

## Graphical abstract

Nitrous oxide (N_2_O) is a potent greenhouse gas with a global warming potential of 310. A novel mechanochemical strategy is presented for decomposing N_2_O into harmless nitrogen and oxygen, achieving a rapid decomposition rate of 1761.3 mL h^−1^ and nearly complete N_2_O conversion of 99.98% at 42 °C.

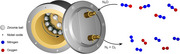

## 1 Introduction

Nitrous oxide (N_2_O), also known as laughing gas, is widely recognized as one of the three major greenhouse gases, alongside carbon dioxide and methane.^[^ ^1^, ^2^ ^]^ Among them, N_2_O poses the most severe global warming potential with a value of 310 (ref. [3]) due to its strong infrared absorption and long atmospheric lifetime of 120 years.^[^ ^4^ ^]^ Moreover, N_2_O is also known to contribute to stratospheric ozone depletion,^[^ ^5^ ^]^ adding to its overall environmental impact. Since 1750, human activities, including agriculture, fossil fuel combustion, and industrial processes, have significantly increased anthropogenic N_2_O emissions, which are projected to be nearly doubled by 2050 (refs. [6, 7]). Climate scientists have recently raised concerns about the abnormal growth in N_2_O emissions and emphasized the urgent need for sustainable solutions to mitigate global warming.^[^ ^8^ ^]^

Various technologies have been proposed to reduce N_2_O emissions,^[^ ^9^, ^10^, ^11^, ^12^ ^]^ and among them, the thermocatalytic N_2_O decomposition is considered one of the most acceptable approaches. While it is effective at high temperatures, its efficiency drops significantly under mild temperature conditions. Although the N_2_O decomposition is thermodynamically spontaneous^[^ ^13^ ^]^ because of its exothermic nature (N_2_O ⇌ N_2_ + 1/2O_2_, Δ*H* = −82 kJ mol^−1^),^[^ ^14^, ^15^ ^]^ its stable resonance structure kinetically prevents the reaction under mild temperature conditions.^[^ ^16^ ^]^ As a result, thermocatalytic N_2_O decomposition requires substantial energy input to raise and maintain high temperatures for kinetic activation of mild‐temperature exhaust gases, as per the Brønsted‐Evans‐Polanyi relation.^[^ ^17^, ^18^ ^]^ To address this issue, a complementary novel method that can operate effectively under mild conditions is needed for efficient and adaptable N_2_O mitigation across diverse emission sources with different exhaust gas temperatures.

Mechanochemistry, considered a fourth wave of chemistry alongside conventional thermochemistry (heat), photochemistry (light), and electrochemistry (electricity), initiates chemical reactions through dynamic mechanical actions, such as collisions, abrasion, friction, rubbing, breaking, and so on. It has shown the potential to overcome limitations associated with conventional chemistry^[^ ^19^, ^20^, ^21^ ^]^ and has recently attracted significant attention as a promising approach to tackle kinetic limitations.^[^ ^22^, ^23^ ^]^ This is because mechanochemically induced thermodynamic non‐equilibrium states play a positive role in enhancing reaction rates under mild conditions.^[^ ^22^, ^24^ ^]^ In addition, mechanochemistry is usually a solvent‐free and waste‐free green process^[^ ^25^ ^]^ which perfectly aligns with the objectives of a sustainable society.

In this work, we explored the potential of mechanochemistry as a complementary approach for N_2_O decomposition. Using a nickel oxide (NiO) catalyst, the mechanochemical N_2_O decomposition achieved a reaction rate of 1761.3 mL h^−1^, higher than that observed in the thermocatalytic method (294.9 mL h^−1^). Notably, the mechanochemical N_2_O decomposition showed an ultimate conversion of 99.98% near ambient temperature of 42 °C, while the thermocatalytic method reached 49.16% at 445 °C. These results highlight the distinct advantages of mechanochemistry under mild conditions. Under dynamic mechanochemical conditions, the surface of the NiO catalyst is continuously refreshed and transformed into an ultra‐oxidized state with a high density of defects induced by dynamic mechanical actions, and this plays a pivotal role in accelerating the N_2_O decomposition.

## 2 Results and Discussion

### 2.1 Mechanochemical N_2_O Decomposition

**Figure 1:**
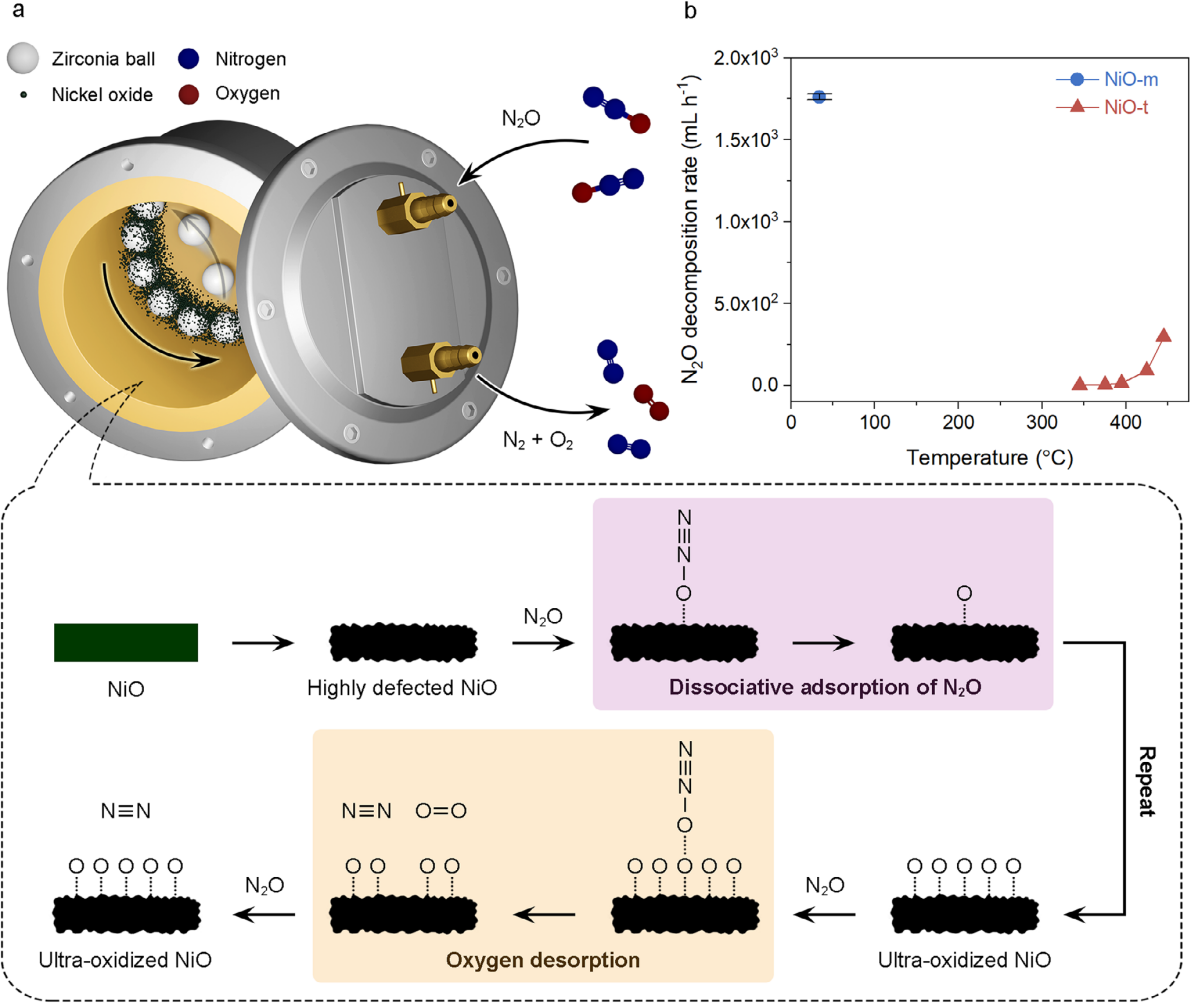
Mechanochemical N_2_O decomposition. a) Schematic representation of the mechanochemical N_2_O decomposition. b) Rates of N_2_O decomposition using mechanochemical and thermochemical methods as a function of operating temperature. The error bars represent the standard deviation obtained from at least five independent experiments.

The mechanochemical N_2_O decomposition process is schematically presented in **Figure** **1**a. The detailed experimental procedures are described in the Supporting Information. NiO was selected as the main catalyst for mechanochemical N_2_O decomposition because of its favorable intrinsic activity. It exhibits a lower activation energy (95.5 kJ  mol^−1^) compared to other bare metal oxides, which is attributed to its ability to efficiently adsorb reactants and desorb products.^[^ ^26^ ^]^ Furthermore, the effect of activation energy on the reaction rate becomes more pronounced at lower temperatures according to the Arrhenius equation ($k=Ae^{-\frac{E_{a}}{RT}})$. These characteristics make NiO a suitable candidate for mechanochemical processes operating under mild temperature conditions.

The surface of the NiO was transformed into a highly defective state by the mechanochemical operation. We expected that this unusual surface transformation would be associated with the powerful impact force of the mechanochemistry process, generated by high‐speed balls. The kinetic energy was transmitted to the NiO and created a high level of defects, which were regenerated by repeated dynamic mechanical actions.

The N_2_O decomposition mechanism proceeds via two main steps.^[^ ^27^, ^28^, ^29^ ^]^ First, N_2_O is adsorbed on the NiO surface and dissociates into nitrogen gas and chemisorbed atomic oxygen (N_2_O → N_2_ + O*). Defect sites with low coordination numbers exhibit high activity, facilitating the dissociative adsorption of N_2_O and thereby accelerating the first step. As the reaction proceeds, the NiO surface becomes progressively enriched with chemisorbed atomic oxygen species (O*), leading to the formation of an ultra‐oxidized state. In the subsequent step, the chemisorbed oxygen species react with additional molecular N_2_O to produce N_2_ and O_2_ (O* + N_2_O → O_2_ + N_2_). The ultra‐oxidized NiO surface promotes efficient oxygen desorption, enabling the reaction to proceed effectively under mild conditions.

All experimental data for NiO in this study were collected after achieving stable catalytic performance, as the catalysts underwent in situ surface transformations during the mechanochemical N_2_O decomposition process induced by ball milling (Figure S1, Supporting Information).

Prior to the in‐depth investigation with the catalysts, a blank test was also meticulously conducted. The reactor was filled with argon gas instead of N_2_O to assess potential contamination of oxygen or nitrogen during the mechanochemical N_2_O decomposition process. The results of the blank test showed that the amounts of nitrogen and oxygen produced were negligible (Figure S2, Supporting Information), affirming contamination‐free reaction conditions.

Catalytic performance of several bare metal oxides (Fe_2_O_3_, Co_3_O_4_, NiO, and CuO) was evaluated (Figure 1b; Figures S3 and S4, Supporting Information, and **Table** **1**). Among them, NiO exhibited the best performance, as anticipated. Notably, the maximum reaction rate of the mechanochemical N_2_O decomposition using NiO catalyst (NiO‐m) reached 1761.3 mL h^−1^ at 34 °C (Figure 1b), which was higher than that of the conventional thermochemical method using the same NiO catalyst (NiO‐t) at 445 °C (294.9 mL h^−1^). To ensure a comprehensive comparison, the N_2_O decomposition rates of the mechanochemical and thermochemical processes were evaluated using various methods (Tables S1 and S2, Supporting Information).

**Table 1:** Mechanochemical N_2_O decomposition rate of several metal oxides.

Sample	Fe_2_O_3_	Co_3_O_4_	NiO	CuO
Decomposition rate [mL h^−1^]	32.3	17.2	1761.3	11.9

Additionally, the mechanochemical method achieved a nearly complete conversion (99.98%) of N_2_O at a mild temperature of 42 °C, compared to the conventional thermochemical process (49.16% at 445 °C) (Figures S5 and S6, Supporting Information). This result is attributed to the unique characteristics of mechanochemistry, which enable it to overcome the high activation energy barrier even at low temperatures.

### 2.2 Parameter Studies

**Figure 2:**
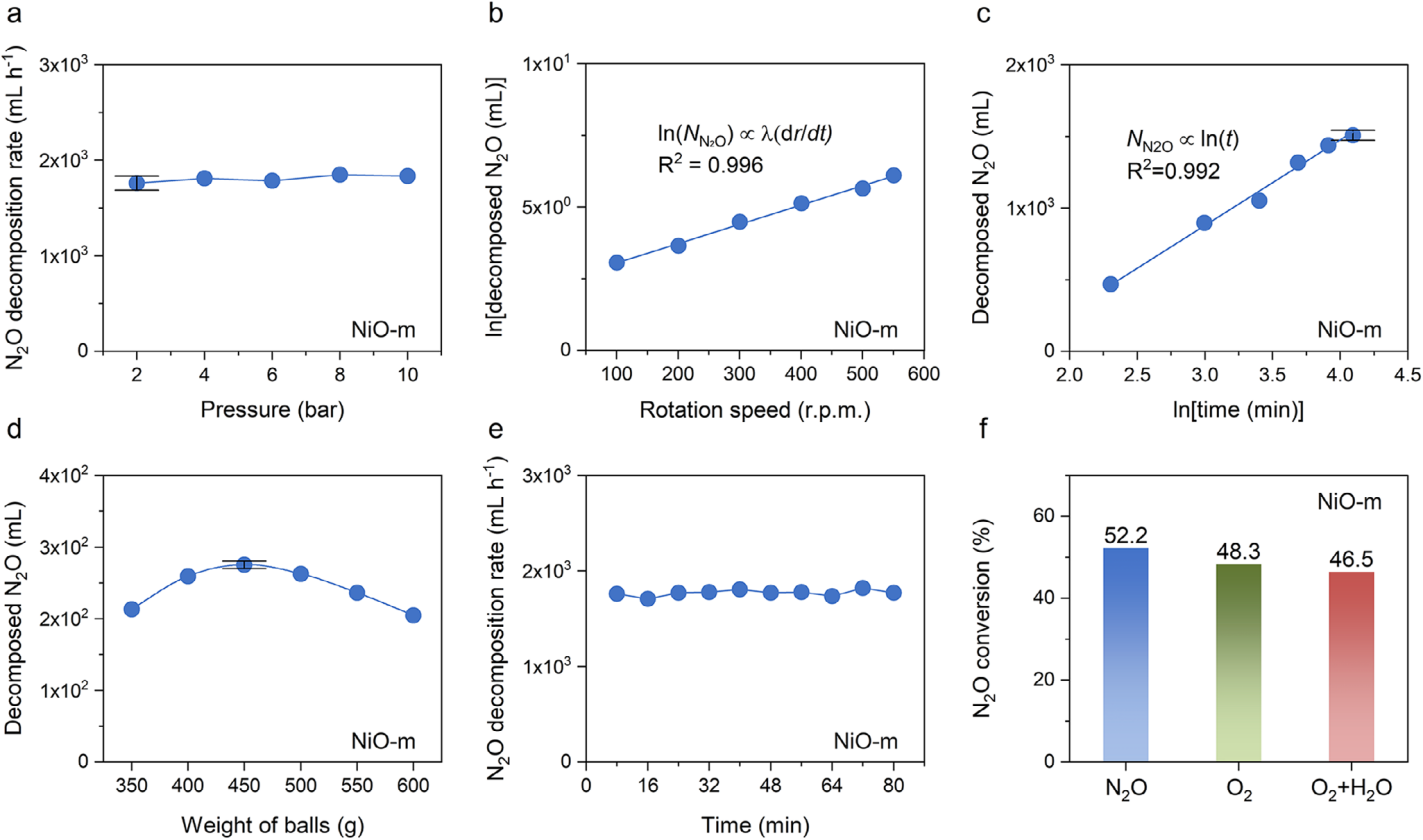
Studies on mechanochemical parameters (pressure, rotation speed, time, ball loading amount, sustainability, and inhibitors). a) Dependence on initial N_2_O pressure. The decomposition rate was independent of the initial pressures. b) Effect of rotation speed on N_2_O decomposition. The total number of rotations for each experiment was fixed at 6600. The N_2_O decomposition performance exhibited a logarithmic correlation with rotation speed. c) Effect of milling time on N_2_O decomposition. The decomposed amount of N_2_O showed a logarithmic correlation with time. d) Effect of ball mass on N_2_O decomposition. The error bars represent the standard deviation obtained from a minimum of five independent experiments. e) Sustainability of the mechanochemical N_2_O decomposition for 10 cycles, using the same NiO catalyst under N_2_O (2 bar) atmosphere for 8 min per experiment. f) Potential inhibitors, including 10% O_2_ alone and a combination of 10% O_2_ and 10% H_2_O in batch mechanochemical N_2_O decomposition.

The relationship between experimental parameters and catalytic performance was further studied to better understand N_2_O decomposition (**Figure** **2**; Figure S7, Supporting Information). The decomposition rate (Figure 2a) and N_2_O conversion (Figure S8, Supporting Information) remained constant with respect to N_2_O pressure. This result could be attributed to two underlying factors. First, the N_2_O decomposition (2N_2_O → 2N_2_ + O_2_) is a volume‐expanding process. According to the equilibrium law, the forward reaction is hindered with pressure increases. Second, as the pressure increases, the concentration of reactants rises while the number of catalytic active sites remains constant, thereby accelerating the forward reaction. These two opposing contributions to the N_2_O decomposition balance each other. As a result, the overall rate of N_2_O decomposition, depending on pressure, followed a zero‐order (pressure independent) reaction.

The amount of decomposed N_2_O (*N*_N2O_) exhibited a natural logarithmic relationship with rotation speed [ln(*N*_N2O_) ∝ *λ*(d*r*/d*t*), (*λ* = 0.67 × 10^−2^)] (Figure 2b). This is because the kinetic energy generated by the milling balls is directly related to the rotation speed and serves as the driving force for catalytic transformation (defect generation and oxidation) and chemical reaction (N_2_O decomposition). A high rotation speed not only supplies sufficient kinetic energy for these processes but also induces a transient local temperature rise due to dynamic mechanical actions.^[^ ^30^ ^]^ However, the overall bulk temperature increase is not significant. Another noteworthy observation was that the mechanochemical N_2_O decomposition could be driven even at a low rotation speed of 100 rpm.

The extent of N_2_O decomposition increases at a progressively diminishing rate over time, exhibiting a linear correlation with the natural logarithm of time [*N*_N2O_ ∝ ln(*t*)] (Figure 2c). This trend is attributable to the reduced reactant concentration as the reaction proceeds, leading to a corresponding decrease in the reaction rate.

The amount of decomposed N_2_O exhibited characteristic volcano‐shaped trends with both the loading amounts of balls (diameter: 3 mm) (Figure 2d) and the ratios of differently sized balls (diameters: 3 and 5 mm) (Figure S9, Supporting Information), at a fixed rotation speed of 550 rpm for 15 min. These trends are attributed to two key variables: the kinetic energy of collisions and the number of collisions per unit time. In the case of ball loading, the collision energy per ball decreases with increasing ball loading due to the reduced travel distance between balls. In contrast, the number of collisions increases as the ball loading increases.

The decrement in catalyst loading resulted in a proportional decline in N_2_O conversion, underscoring the pivotal influence of catalyst amount for the efficiency of mechanochemical N_2_O decomposition (Figure S10, Supporting Information).

The sustainability of the mechanochemical method was further substantiated through ten consecutive reaction cycles conducted over a total of 80 min (Figure 2e), confirming the robustness of the mechanochemical approach. Each cycle was carried out under a 2 bar N_2_O atmosphere for 8 min.

To assess the impact of O_2_ and H_2_O as potential inhibitors, control experiments were performed using a planetary batch ball mill system. The reaction conditions were modified by introducing 10% O_2_ alone and a combination of 10% O_2_ and 10% H_2_O (Figure 2f). Both O_2_ and H_2_O marginally affected the reaction kinetics, suggesting that the dynamic mechanochemical N_2_O decomposition was not significantly affected by reaction environments.

Based on collective operation parameters, including pressure independence, mild reaction conditions, sustainability, and inhibitors suggested the practicality of the mechanochemical N_2_O decomposition process for potential real‐world applications (vide infra).

### 2.3 Catalyst Characterizations

**Figure 3:**
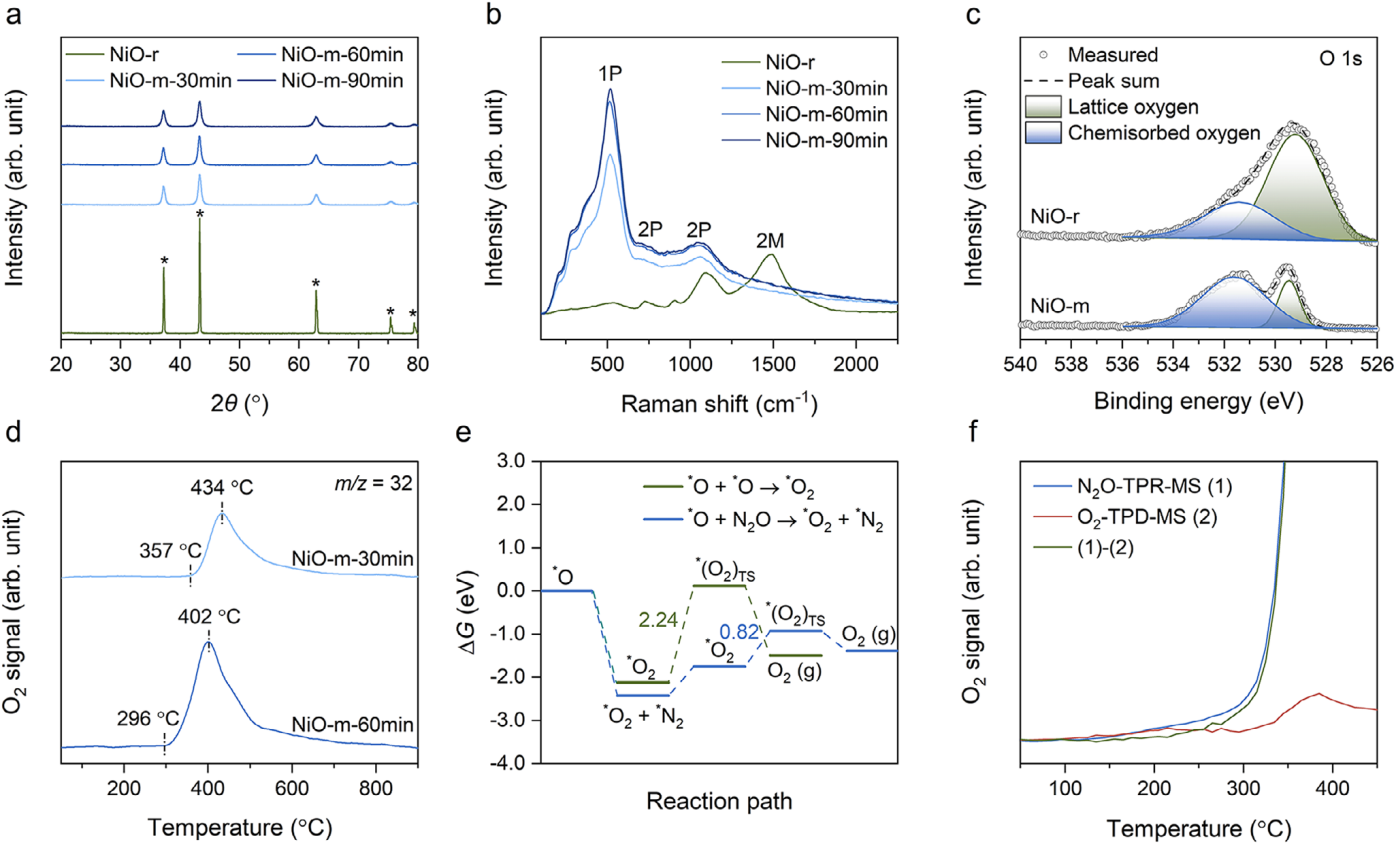
Catalyst characterizations. a) XRD patterns of pristine (NiO‐r, reference) and post‐processed NiO samples (NiO‐m, mechanochemical treatment) depending on the ball milling time for 30, 60, and 90 min under N_2_O atmosphere. The peaks labeled with asterisks indicate the pristine crystalline NiO powder (PDF No. 47‐1049). b) Raman spectra of NiO samples. c) XPS spectra of NiO catalysts focusing on the O 1s binding regions. d) O_2_‐TPD‐MS curves of NiO samples depending on the ball milling time. O_2_‐TPD‐MS profile was measured using BELCAT II (MicrotracBEL) after He pre‐treatment at 50 °C for 1 h, followed by ramping up to 900 °C at 10 °C min^−1^ under He flow (30 mL min^−1^). e) DFT calculations of the oxygen desorption step on defect‐free NiO(200). f) O_2_ signal of N_2_O‐TPR‐MS, O_2_‐TPD‐MS and their subtraction on NiO‐m‐60 min. O_2_ signal was obtained using a custom fixed‐bed reactor under identical conditions: Ar flow (10 mL min^−1^), temperature ramping from room temperature to 450 °C at 2 °C min^−1^, and O_2_ signal detection by GC every 5 min.

Catalyst characterizations were conducted to investigate structural and chemical changes, and their impact on catalytic performance. Powder X‐ray diffraction (XRD) analyses of the NiO‐r (pristine NiO reference) and post ball‐milled NiO samples for different durations under N_2_O atmosphere (NiO‐m‐30 min, NiO‐m‐60 min, NiO‐m‐90 min) revealed significant structural transformations during ball milling process (**Figure** **3**a). The NiO used for the dynamic mechanochemical N_2_O decomposition (NiO‐m) showed broader and weaker peaks than those of NiO‐r, indicating that its crystallinity decreased because of the high density of defects.

Raman spectroscopy further supported the structural change of NiO during ball milling under N_2_O atmosphere (Figure 3b). The intensity of the 1P peak at 512 cm^−1^, typically inactive in NiO‐r, increased markedly with milling time, indicating the formation of structural defects such as oxygen vacancies and low‐coordinated Ni sites that disrupted crystal symmetry. The 2P peak at ≈1100 cm^−1^ exhibited broadening with increased milling time, reflecting enhanced lattice disorder induced by mechanical strain and defect generation. Furthermore, the 2M peak at 1490 cm^−1^, characteristic of antiferromagnetic ordering, disappeared after prolonged milling, suggesting that oxygen vacancies disrupted spin exchange interactions.^[^ ^31^ ^]^

The monitored structural evolution by XRD and Raman spectroscopy indicates that significant changes occur during the early stages of milling, but these transformations gradually diminish and tend to reach a plateau between 60 and 90 min. This result suggests that the catalyst reaches a steady state after a certain milling duration because of a dynamic equilibrium between comminution and reconstruction in the mechanochemical system.^[^ ^23^ ^]^ This trend aligns well with the N_2_O decomposition performance, implying that structural saturation correlates with stable catalytic behavior (Figure S11, Supporting Information).

The high degree of defects in NiO‐m was also confirmed by electron paramagnetic resonance (EPR) measurements (Figure S12, Supporting Information), showing an increase in the *g*‐factor from 2.542 to 3.855 and broadening of the signal with extended ball milling time. These changes are attributable to the formation of low‐coordination structural defects, such as oxygen and Ni vacancies, which serve as localized traps for unpaired electrons. These defects enhance exchange interactions between the trapped electrons and neighboring Ni^2+^ ions, altering the local magnetic environment and increasing the *g*‐factor. The signal broadening is a result of the random distribution of defects, which intensifies spin‐spin interactions and leads to inhomogeneous broadening.^[^ ^32^ ^]^

Transmission electron microscopy (TEM) observations corroborated these findings (Figure S13, Supporting Information). High‐resolution TEM (HR‐TEM) images and profiles of inverse fast Fourier transition (IFFT) indicated an amorphous morphology on the surface of NiO‐m, because of the abundant number of defects generated. Particle size distribution and transformation process were also examined with TEM analysis (Figure S14, Supporting Information). The particle size of NiO decreases rapidly during the initial stages of milling and gradually approaches saturation, indicating it cannot decrease indefinitely with extended ball milling time. This trend is consistent with the results of XRD and Raman spectroscopy (Figure 3a,b).

The advantage of highly defective NiO‐m for the dissociative adsorption of N_2_O was investigated through control experiments (Figure S15, Supporting Information). Specifically, pristine NiO was first ball‐milled and then exposed to N_2_O without mechanical agitation. Even under static conditions, the first dissociative adsorption step (Figure 1a) occurred. This was attributed to the presence of low‐coordinated defect sites, which were highly active and promoted the dissociative adsorption of N_2_O.^[^ ^33^, ^34^ ^]^

Quantitative optical analyses were also carried out using ultraviolet‐visible (UV‐vis) spectroscopy (Figure S16, Supporting Information). The NiO‐m sample was diluted 2‐fold with polytetrafluoroethylene (PTFE) due to its relatively strong absorbance. Despite the dilution, it still exhibited strong absorption peaks in the broad range of 500–600 nm, indicating a high density of non‐stoichiometric Ni and O ratio in NiO‐m (NiO_x_, x ≠ 1).^[^ ^35^, ^36^ ^]^ Two broad peaks of NiO‐m ≈420 and 725 nm, associated with the *d‐d* transition of Ni^2+^ ions in the crystalline phase of NiO,^[^ ^37^, ^38^ ^]^ became almost flat, whereas these peaks were clearly observed in NiO‐r. This result further supports the non‐stoichiometric Ni and O ratio in NiO‐m. The near‐flat peak shape observed from NiO‐m was a consistent characteristic across all diluted NiO‐m samples (Figure S17, Supporting Information). Visual color changes also provided supporting evidence for the UV‐vis findings. The color of the NiO samples shifted from green (NiO‐r) to black (NiO‐m) as the proportion of non‐stoichiometric Ni and O ratio increased (inset, Figure S16, Supporting Information).^[^ ^39^ ^]^ This observation suggests that the significant deviation from stoichiometry in NiO‐m was induced by the dynamic mechanochemical process.

The X‐ray photoelectron spectroscopy (XPS) results of the O 1s spectra provided insights into the surface oxidation states (Figure 3c). The NiO‐m sample exhibited distinct spectra compared to NiO‐r. The deconvoluted peaks at 529.2 and 531.4 eV correspond to lattice oxygen and chemisorbed oxygen species, respectively.^[^ ^40^ ^]^ These observations suggest that a significant portion of NiO‐m underwent a highly oxidized transformation during the mechanochemical process. Furthermore, NiO‐m exhibited a higher surface oxygen concentration (74.9%) than both commercial NiO‐r (65.8%) and even Ni_2_O_3_ (66.7%) (Table S3, Supporting Information), implying a high density of chemisorbed oxygen species on its surface.

The detailed oxidation states of the samples were further elucidated using electron energy loss spectroscopy (EELS) (Figure S18, Supporting Information). The ratio of Ni L_3_/L_2_ exhibited an inverse correlation with the oxidation state of Ni.^[^ ^41^, ^42^ ^]^ Notably, NiO‐m showed a significantly lower ratio than the commercial NiO‐r and Ni_2_O_3_. The collective interpretation together with the preceding characterizations indicates that the surface of NiO‐m was more highly oxidized than even that of commercial Ni_2_O_3_.

Ultra‐oxidized nickel oxide (Ni_x_O_y_, x = 2, y > 3) is unstable due to its lower formation energy compared to stoichiometric NiO (Figure S19, Supporting Information). However, the unique non‐equilibrium state driven by the dynamic mechanochemical actions enables the formation of ultra‐oxidized NiO‐m, in which atomic oxygen (O*) is chemisorbed on the surface.

Oxygen temperature‐programmed desorption mass spectrometry (O_2_‐TPD‐MS) offered additional understanding into the performance of the ultra‐oxidized NiO‐m during the second O_2_ desorption step (Figure 3d). A significant amount of desorbed oxygen observed in NiO‐m samples indicated that the mechanochemical process generated ultra‐oxidized NiO‐m with high surface oxygen coverage. The adsorbed oxygen was mainly attributable to atomically chemisorbed oxygen species (O*).^[^ ^43^ ^]^ The notable observation was that NiO‐m exposed to N_2_O atmosphere for 60 min (NiO‐m‐60 min) exhibited faster oxygen desorption than the sample treated for 30 min (NiO‐m‐30 min). This result suggests that the ultra‐oxidized NiO‐m facilitates the oxygen desorption step due to the weakened binding energy of chemisorbed oxygen.^[^ ^44^ ^]^ The strong repulsive interactions caused by high oxygen density on the NiO‐m surface promote the efficient desorption of molecular oxygen (O_2_). In other words, the ultra‐oxidized state of NiO‐m plays a key role in facilitating the effective oxygen desorption step under mild conditions.

To further investigate the mechanism of mechanochemical N_2_O decomposition, density functional theory (DFT) calculations were performed. Based on previous reports,^[^ ^6^, ^45^ ^]^ the N_2_O decomposition proceeds via two steps. In the first step, N_2_O dissociates into N_2_ and chemisorbed atomic oxygen (O*) on the catalyst surface. The DFT results show that N_2_O preferentially adsorbs on defective sites (Figures S20 and S21, Supporting Information), thereby facilitating its activation.^[^ ^46^ ^]^ Defect sites exhibit high adsorption energy toward O*, up to −3.03 eV, which means that the O* can be accumulated on the NiO surface (Figure S21, Supporting Information). This result explains why NiO‐m is enriched with O*.

The second step is the desorption of as‐dissociated O* from the catalytic surface into molecular oxygen (O_2_).^[^ ^47^, ^48^, ^49^ ^]^ Two possible pathways are proposed for this step: (1) O* + N_2_O → O_2_ + N_2_ and (2) O* + O* → O_2_. DFT calculations suggest that the energy barrier for the first pathway is significantly lower (0.82 eV) than that of the second pathway (2.24 eV), as shown in Figure 3e. This difference arises from the distinct adsorption configurations of the intermediate *O_2_ species. In the second pathway (O* + O* → O_2_), *O_2_ is adsorbed on the catalyst surface through two oxygen atoms to form a bidentate ligand in a side‐on configuration (Figure S22, Supporting Information). In contrast, the *O_2_ in the first pathway (O* + N_2_O → O_2_ + N_2_) is adsorbed through one oxygen atom to form a monodentate ligand in an end‐on configuration, which is comparatively less stable.

To provide clear experimental evidence for the step 2 mechanism (O* + N_2_O → O_2_ + N_2_), control experiments were performed using N_2_O temperature‐programmed reaction mass spectrometry (N_2_O‐TPR‐MS) and O_2_‐TPD‐MS on the post‐milled NiO sample under N_2_O conditions (NiO‐m‐60min, Figure 3f). In the O_2_‐TPD curve, desorption peaks corresponding to physisorbed O_2_ appeared below 300 °C, while desorption associated with the O* + O* → O_2_ recombination pathway was observed above ≈300 °C.^[^ ^43^, ^50^, ^51^, ^52^ ^]^ To isolate the effect of chemisorbed species, the N_2_O ‐TPR curve was subtracted from the O_2_‐TPD curve, excluding the contribution from physisorbed O_2_. The resulting spectrum showed a significant O_2_ signal emerging ≈300 °C, where desorption via the O* + O* recombination is unlikely. This observation indicates that chemisorbed O* can desorb through a faster pathway than the O^*^ + O^*^ → O_2_ recombination, providing direct experimental evidence for O* + N_2_O → O_2_ + N_2_ as a viable reaction pathway. To further assess the contribution of O* + O* → O_2_ and O^*^ + N_2_O → O_2_ + N_2_ pathways for step 2, another control experiment was conducted by short milling oxygen‐preadsorbed NiO under Ar or N_2_O conditions. The resulting O_2_ volumes provide experimental support for the dominant role of the O^*^ + N_2_O → O_2_ + N_2_ pathway (Figure S23, Supporting Information). Altogether, both theoretical and experimental results suggest that the accumulated O* of the ultra‐oxidized NiO surface also functions as an active site for further N_2_O decomposition. Moreover, additional control experiments confirmed that step 2 is the rate‐determining step (RDS) in the overall mechanochemical N_2_O decomposition (Figure S24, Supporting Information). This result indicates that improving oxygen diffusion kinetics through the ultra‐oxidized phase of NiO is a main factor in enabling efficient N_2_O decomposition under mild conditions.

On the other hand, the particle size distribution and textural properties of the size‐reduced catalyst (NiO‐sr) before and after mechanochemical and thermochemical reactions were compared. The result indicated that the thermochemical process causes a significant loss of catalytic activity by aggromeration at high temperatures, while the mechanochemical process effectively preserves catalyst stability (Figures S25, S26, and Table S4, Supporting Information).

It is noteworthy that the active sites on the NiO surface exhibit structural self‐regulation, with N_2_O dissociation occurring on defective sites and O_2_ desorption favoring extended surfaces. The surface of the catalyst undergoes dynamic reconstruction, driven by Ostwald ripening, coalescence, and mechanical force‐induced comminution. Fast mass diffusion and surface reconstruction create the possibility for continuously regenerated active sites to be involved in the dissociative adsorption of N_2_O and the desorption of O_2_.

In addition to the surface changes on the NiO catalyst, other factors contribute to the outstanding performance of the mechanochemical method under mild temperatures. The dynamic kinetic energy generated by the mechanical actions serves as the driving force for the N_2_O decomposition. The repeated dynamic mechanical actions accelerate the desorption of intermediates,^[^ ^23^ ^]^ such as the molecular oxygen desorption step. Moreover, the mechanochemical actions generate local heat, which can reach temperatures as high as 700 °C within milliseconds.^[^ ^53^ ^]^ Although this is not significantly responsible for the bulk reaction temperature increases, this local heat might also contribute to overcoming the activation barrier for N_2_O decomposition.

### 2.4 Practicality Evaluations

**Figure 4:**
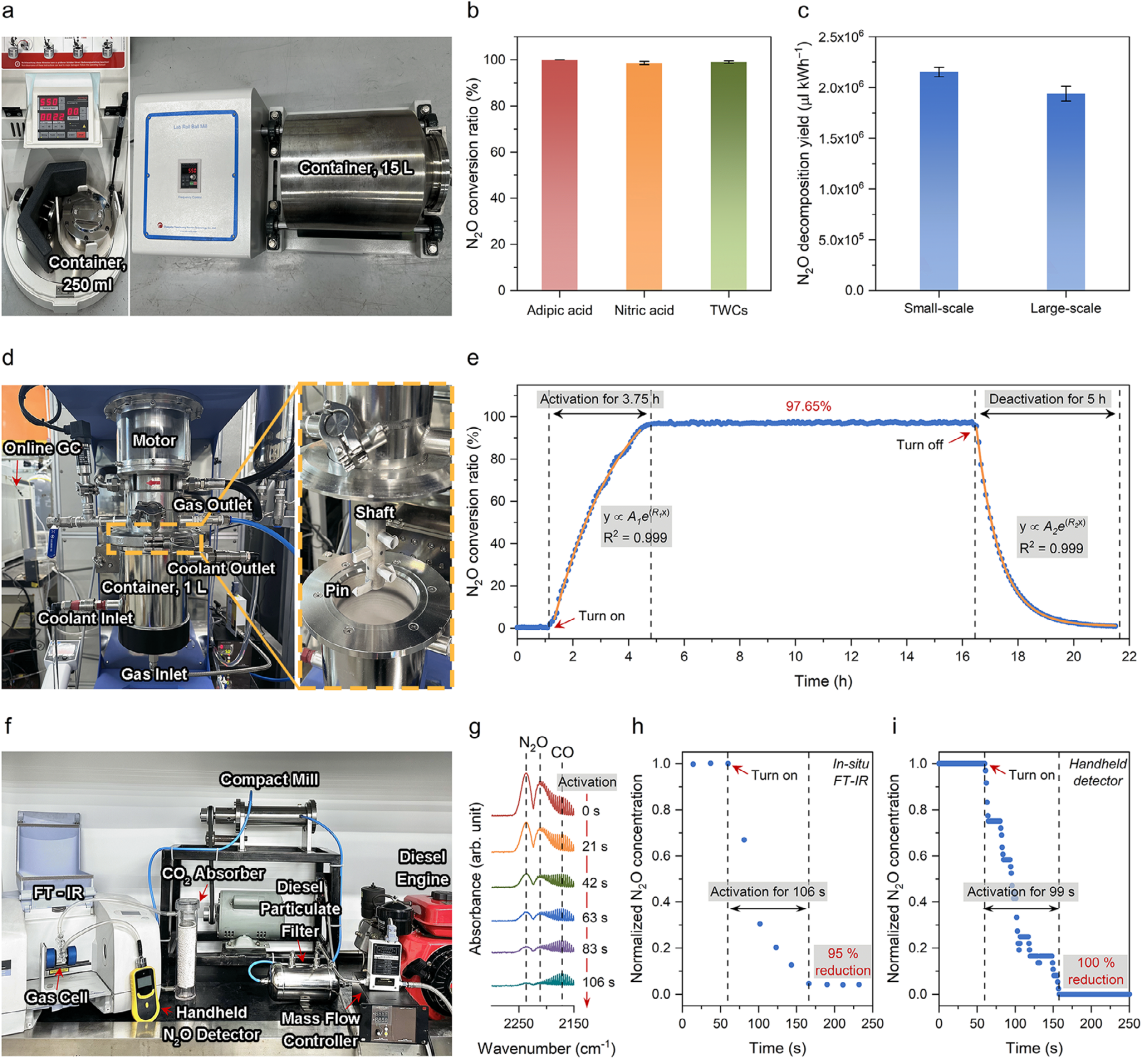
Practicality of the mechanochemical N_2_O decomposition. a) Photograph of planetary mill with container of 250 mL (left) and roll‐mill with container of 15 L (right). b) N_2_O conversion of the mechanochemical method using gas mixtures with representative exhaust gas compositions emitted by real industrial processes. c) Energy efficiencies of small and large‐scale mechanochemical N_2_O decomposition. The error bars represent the standard deviation of three independent experiments. d) Photograph of a continuous milling system based on a customized attrition‐mill (left) and enlarged structure of a rotating shaft with pins (right). Mechanochemical force to drive the reaction is generated by the collisions between the ZrO_2_ balls and pins on the rotating shaft. e) Change in N_2_O conversion depending on the continuous milling system under atmospheric pressure at a constant reactor temperature of 25 °C controlled with a water chiller. f) Photograph of a homemade MCR system for exhaust gas treatment from a diesel engine. g) In situ FT‐IR spectra of exhaust gases from a diesel engine. The activation time corresponding to each spectrum was represented with a red arrow. h) Normalized N_2_O concentration detected by in situ FT‐IR spectra, showing that the continuous N_2_O reduction with the MCR system reached ≈95% after mechanochemical NiO activation for 106 s. i) Normalized N_2_O concentration detected by a handheld N_2_O detector showing that the continuous N_2_O reduction with the MCR system reached 100% after mechanochemical NiO activation for ≈99 s.

Exhaust gases that contain different compositions of N_2_O are generated by various sources.^[^ ^8^ ^]^ Therefore, it is important to demonstrate the versatility of the mechanochemical method with diverse exhaust gases produced by industrial processes. Mixture gases with similar compositions to real exhaust gases from representative sources of N_2_O, such as nitric acid production, adipic acid production, and the three‐way catalyst (TWC) process, were used in the mechanochemical N_2_O decomposition system (Table S5, Supporting Information). Under the same experimental conditions with the planetary mill (**Figure** **4**a, left), mechanochemistry achieved almost complete N_2_O conversion with these gases (Figure 4b). The mechanochemistry method also demonstrated good stability during repeated cycles with real exhaust gases, even in the presence of other compounds, including NO_x_, O_2_, and CO, which might potentially poison the active sites of NiO catalysts (Figure S27, Supporting Information).

For scaling up the mechanochemical process, a large roll‐mill is a suitable model (Figure 4a, right). The specific operation of the roll‐mill is described in Supporting Information and Figure S28 (Supporting Information). A blank test was also conducted to ensure the system was operating contamination‐free (Figure S29, Supporting Information). Despite the different energy intensities of the roll‐mill (low) and planetary mill (high), the roll‐mill still performed with good energy efficiency, comparable to that of the planetary mill (Figure 4c). This result is consistent with the observation that the mechanochemical N_2_O decomposition could be achieved over a wide range of mechanical energies, even at low energy levels of 100 rpm, as demonstrated in Figure 2b.

Recently, a system for continuous mechanochemical process received a lot of attention, because it is the most reliable for industry applications.^[^ ^54^, ^55^ ^]^ However, achieving efficient continuous reactions with high conversion and fast reaction rates is still an unexplored area in the mechanochemistry field.

In the present study, continuous mechanochemical N_2_O decomposition was carried out using a customized attrition mill equipped with a 1 L reactor (Figure 4d). The detailed operation of the continuous milling system is presented in Figure S30 (Supporting Information). When the system was turned on, a high N_2_O conversion of 97.65% was achieved (Figure 4e). During operation, the N_2_O decomposition was continuously maintained without performance decay for ≈12 h. The inclining and declining curves after turning the system on and off, respectively, follow an exponential relationship as a function of reaction time [*y* ∝ *A*_1_ *e* ^(^ *^R^* ^1x)^, *y* ∝ *A*_2_ *e* ^(^ *^R^* ^2x)^, (*A*_1_ = −0.21 × 10^3^, *R*_1_ = −0.40 × 10^1^, *A*_2_ = 0.33 × 10^11^, *R*_2_ = −0.12 × 10^1^)]. These gradual transitions were attributed to the large reactor volume (1 L), which prevents instantaneous reflection of the conversion. Moreover, the mechanochemical system requires an induction period to in situ generate the highly oxidized, defective NiO surface necessary to reach maximum conversion. Additionally, mechanochemical N_2_O decomposition exhibited over fourfold higher energy efficiency compared to the thermochemical method in both batch and continuous methods (Table S6, Supporting Information).

To demonstrate the practical potential of mechanochemical N_2_O decomposition, a homemade mechanochemical N_2_O reduction (MCR) device was designed and tested for a real exhaust gas treatment from a diesel engine. Many countries are enforcing regulations on diesel engine emissions. Especially, the recently established Euro VII Emission Standard added N_2_O as a newly regulated substance,^[^ ^56^ ^]^ making it difficult to comply with currently existing technologies. Here, we developed a real‐world system for exhaust gas treatment from a diesel engine (Figure 4f; Video S1, Supporting Information). The Fourier transform infrared (FT‐IR) gas cell was connected to the outlet of the MCR system, and the exhaust gases were analyzed using an in situ FT‐IR spectroscopy. Additional N_2_O gas adjusted by mass flow controller (MFC) was mixed with exhaust gases, because the original N_2_O concentration from the small diesel engine was out of the detection limit for in situ FT‐IR measurements. A medical‐grade CO_2_ absorption column was also installed (Figure S31, Supporting Information), because a strong CO_2_ response interfered with N_2_O signals. The N_2_O peak intensities at 2237 and 2212 cm^−1^ were reduced as low as 95% under continuous exhaust gas flow after an activation period of 106 s (Figure 4g, h).^[^ ^57^ ^]^ This result was double checked using a handheld N_2_O detector (Figure 4i). Reliability of devices used in this work was further confirmed by FT‐IR measurements (Figures S32 and S33, Supporting Information). In addition, other nitrogen oxides (NO_x_), emitted by the diesel engine, were also reduced to ≈70% (Figures S34, Supporting Information). Last but not least, the practical issues, such as catalyst leakage and moisture condensation, may not be notably associated with the MCR system. The ball‐milled catalyst particles with high‐density defects are prone to sticking to each other (Figure S35, Supporting Information),^[^ ^23^ ^]^ preventing leakage under the continuous exhaust gas flow. Furthermore, high‐temperature ceramic filter technologies could be an alternative option to avoid possible catalyst leakage in the real world.^[^ ^58^ ^]^ Regarding the moisture condensation issue, the temperature of diesel exhaust gas is typically controlled at ≈400 °C.^[^ ^59^ ^]^ In addition, local and/or bulk heat generated by mechanical actions could also reduce the possibility of moisture condensation in a real system.^[^ ^53^ ^]^ Nevertheless, we conducted an additional experiment to demonstrate that the condensed water effect was also negligibly impacting the MCR performance (Figure S36, Supporting Information). These overall results suggest that the primitive and yet optimized MCR system holds a promise for potential contribution to resolving real‐life issues.

**Figure 5:**
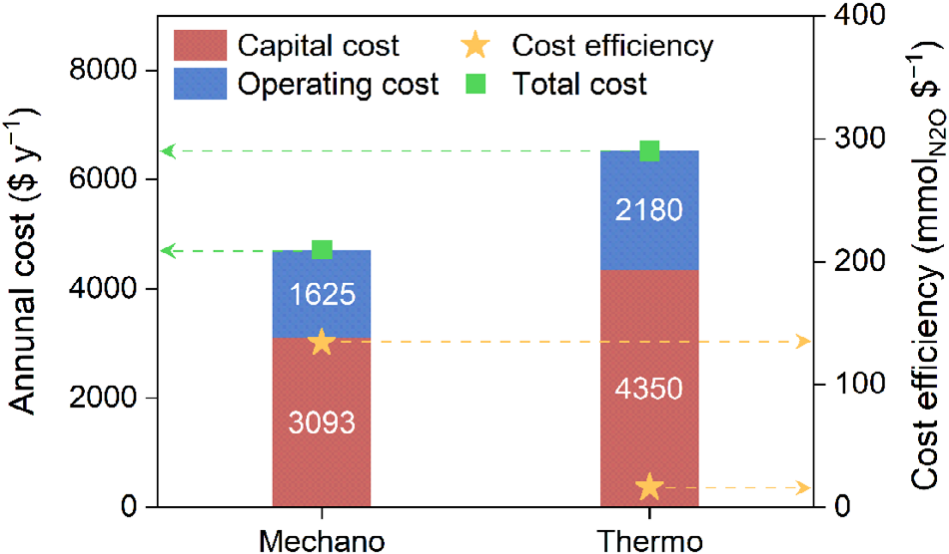
Comparative economic analysis of the mechanochemical and thermochemical methods for N_2_O decomposition. Annual N_2_O decomposition cost and cost efficiency were estimated under consistent assumptions, including 10 year equipment lifetime, 4.5% discount rate, electricity cost of $0.13 kWh^−1^, and 8000 annual operating hours.

A comparative economic analysis was conducted by evaluating the cost efficiency (mmol $^−1^) of two approaches: the mechanochemical and the thermochemical methods (**Figure** **5**). First of all, costs were calculated on an annual basis ($ y^−1^), and thus the cost efficiency could be derived by dividing the annual N_2_O decomposition rate (mmol y^−1^) by the corresponding annual cost (Tables S7 and S8, Supporting Information). To ensure comparability, common assumptions were applied, including the equipment lifetime of 10 years, a discount rate of 4.5%, an electricity cost of $0.13 kWh^−1^, and an annual operation of 8,000 h. Capital costs included the mill, container, milling ball, and catalyst for the mechanochemical method, and the fixed‐bed reactor, quartz tube, and catalyst for the thermochemical method. Supplementary equipment (supplement) costs were assumed to be 20% of the sum of equipment costs (excluding supplement). Operating costs comprised electricity, labor, maintenance, and other costs. Labor costs were assumed to be 3% of the total capital cost, while maintenance and other costs were assumed to be 2% and 1%, respectively, of the main equipment cost (excluding catalyst and supplement). Based on the calculated annual costs and decomposition rates for each method, the cost efficiency of the mechanochemical method was 134.14 mmol $^−1^, significantly higher than that of the thermochemical method, which was 16.23 mmol $^−1^. These results implied that the mechanochemical approach could be a more cost‐effective alternative for N_2_O decomposition, given its favorable decomposition capacity per dollar spent.

## 3 Conclusion

We developed a mechanochemical method to efficiently decompose environmentally hazardous N_2_O under mild conditions. While previous mechanochemical reactions have predominantly focused on chemical syntheses,^[^ ^22^, ^23^, ^60^ ^]^ this approach could be the first application of mechanochemistry to decompose one of the top three global warming gases. Using various characterization techniques, we verified the origins of the mechanochemical N_2_O decomposition. In addition, numerous practicality evaluations suggested its versatility and scalability beyond academic research.

## Conflict of Interest

The authors declare no conflict of interest.

## Supporting information



Supporting Information

Supplemental Video 1

## Data Availability

The data that support the findings of this study are available from the corresponding author upon reasonable request.
