# Supplementary material for: Mechanochemical Nitrous Oxide Decomposition
Source: Adv Mater. 2025 Sep 26;38(2):e11666. doi: 10.1002/adma.202511666 (PMC12783976; doi:10.1002/adma.202511666)
Supplement: Supplementary file 1 — Supporting Information [file ADMA-38-e11666-s002.docx]

**Supporting Information**

**Mechanochemical Nitrous Oxide Decomposition**

Seung-Hyeon Kim, Li-Bo Chen, Jae Seong Lee, Ayeon Kim, Jeong-Min Seo, Jae-Hoon Baek, Se Jung Lee, Boo-Jae Jang, Changqing Li, Runnan Guan, Yanhua Shao, Jian Li, Xing-You Lang, Yung Sam Kim, Gao-Feng Han,* Qing Jiang,* and Jong-Beom Baek*

**Table of Contents**

**Methods: 3**

Mechanochemical N_2_O decomposition 3
Pressure dependence on the mechanochemical N_2_O decomposition 3
Rotation speed dependence on the mechanochemical N_2_O decomposition 3
Milling time dependence on the mechanochemical N_2_O decomposition 3
Ball loading weight and ratio dependence on the mechanochemical N_2_O decomposition 3
Quantification of N_2_O decomposition 4
Thermocatalytic N_2_O decomposition 4
Mechanochemical N_2_O decomposition with exhaust gases 4
Scale-up test using large roll-mill 5
Continuous system using attrition-mill 5
Diesel engine exhaust gas treatment using compact mill 5
Characterizations 5
Theoretical calculation 6

**Figures: 8**

**Tables: 44**

**Video: 52**

**References: 53**

**Methods:**

To begin with, all the data presented in this work were obtained after achieving stable catalytic performance, because ball-milling causes the catalysts to undergo *in-situ* surface transformation during the mechanochemical N_2_O decomposition.

**Mechanochemical N_2_O decomposition**

Nitrous oxide (N_2_O) decomposition experiments were carried out using a planetary ball-mill machine (Pulverisette 6, Fritsch). The catalytic performances of various materials, including NiO (99%, Alfa Aesar), Fe_2_O_3_ (98.5+%, Duksan), Co_3_O_4_ (99%, Alfa Aesar), and CuO (98%, Daejung), were investigated. The experimental setup involved loading mixed zirconium oxide (ZrO_2_) balls (the ratio between balls of 3 and 5 mm diameters = 1:1, 450 g) along with different catalysts (NiO 24.0 g, Fe_2_O_3_ 26.0 g, Co_3_O_4_ 25.8 g, CuO 26.1 g) into the zirconia container, resulting in a remaining volume of 245 ml after all materials were loaded. To ensure a fair comparison, the loaded catalysts contained the same mole of metal atoms for each experiment. Before introducing the N_2_O gas, the jar was thoroughly evacuated for an hour to remove any physiosorbed contaminant on the surface of system that originated from the air. Subsequently, N_2_O gas (99+%, Pacsun, Korea) was charged into the system at 2 bar, and the gas valve was sealed to build a closed system. The ball-milling process was carried out at 550 rpm for 8 min, while the bulk container temperature was directly measured using an infrared thermometer.

A blank test was also conducted following the same process described above, but charging with argon gas (99.999%, Deokyang, Korea) instead of N_2_O.

During the ball-milling process, only the catalysts in collision regions actively participated in the mechanochemical reaction. The working catalyst was calculated to be 0.08%.

**Pressure dependence on the mechanochemical N_2_O decomposition**

The relationship between N_2_O charging pressure and its decomposition was studied. The experiments were carried out at 2, 4, 6, 8, and 10 bar, while the condition was set up with a rotation speed of 550 rpm for 8, 16, 24, 32 and 40 min. After every 8 min operation, the milling system was paused for 10 min.

**Rotation speed dependence on the mechanochemical N_2_O decomposition**

The influence of ball-milling rotation speed was investigated by fixing the number of rotations (6600 cycles). The rotation speed was varied at 100, 200, 300, 400, 500, and 550 rpm, with corresponding reaction times of 66, 33, 22, 16.5, 13.2, and 12 min, respectively.

**Milling time dependence on the mechanochemical N_2_O decomposition**

Mechanochemical N_2_O decomposition was conducted at a pressure of 8 bar and a rotational speed of 550 rpm, with reaction times of 10, 20, 30, 40, 50, and 60 minutes. To prevent excessive temperature rise during ball milling, a 10 minute pause was introduced after every 10 minutes of operation.

**Ball loading weight and ratio dependence on the mechanochemical N_2_O decomposition**

The number of collisions and the power of the collision impacts were influenced by the loading weight and the ratio of loaded balls. To evaluate the effect of loading weight, mechanochemical experiments were performed under N_2_O (4 bar) at 550 rpm for 15 min, using 3 mm balls of different weights, specifically, 350, 400, 450, 500, 550, and 600 g.

The effect of ball ratio was also studied by mixing the balls with different diameters (3 and 5 mm). These experiments were conducted under N_2_O (4 bar) at 550 rpm for 15 min, with a fixed ball weight of 450 g, while the weight percentages of balls (5 mm) were varied 0, 25, 50, 75, and 100%.

**Quantification of N_2_O decomposition**

Before measuring the gas product of the mechanochemical reaction, argon was flowed through the system for one hour to remove any absorbents on the surface of the gas line. The quantification of decomposed N_2_O was achieved by passing the gas product from the container to an online gas chromatograph (7890B, Agilent) equipped with a specific column (10 ft, 1/8-inch i.d., 2 mm o.d., HayeSep Q 80/100 μm). The peak areas obtained from the gas chromatography represented N_2_, O_2_, and N_2_O. Then, the peaks were converted to the volume fraction of the product gas (*φ*_i_) using calibration plots.

To determine the N_2_O conversion, two methods were adopted for crosscheck. The first method calculated the N_2_O conversion with a formula of [(*φ*_N2_/(*φ*_N2_ + *φ*_N2O_)) * 100]. The second method used the equation of [((*φ*_N2_ * *P*_product_)/*P*_charge_) * 100], where the pressures of the product gas (*P*_product_) and the initially charged N_2_O gas (*P*_charge_) were measured by connecting a pressure meter to the gas valve of the ball-milling jar. Both results of N_2_O conversion were well matched with each other. Finally, the amount of decomposed N_2_O was determined by multiplying the N_2_O conversion with *P*_charge_ and the remaining volume of the ZrO_2_ container (245 ml).

**Thermocatalytic N_2_O decomposition**

Size-reduced NiO (NiO-sr) was prepared under argon gas atmosphere at the same conditions of NiO-m under N₂O atmosphere. In this experiment, this NiO-sr was used for thermochemical N₂O decomposition reaction, ensuring a fair comparison between mechanochemical and thermochemical methods by minimizing surface area difference. Typically, NiO-sr (1.0 g) was loaded in a quartz tube with an inner diameter of 6 mm. Prior to starting the experiments, the tube was purged with argon gas at 20 standard cubic centimeters per minute (SCCM) for 30 min to ensure the removal of any impurity. The fixed-bed reactor was then heated to 445 °C at a ramping rate of 2 °C per min, while the flow rate of N_2_O gas was maintained at 10 SCCM. The other catalysts (Co_3_O_4_, CuO, and Fe_2_O_3_) were also tested using the same procedure.

**Mechanochemical N_2_O decomposition with exhaust gases**

In order to confirm the versatility of the mechanochemical method, mechanochemical N_2_O decomposition experiments were conducted using gas mixture compositions similar to the real exhaust gases generated from nitric acid production, adipic acid production, and three-way catalysts. The experimental procedure was the same as the standard mechanochemical N_2_O decomposition except the gases.

**Scale-up test using large roll-mill**

The scale-up of the mechanochemical method was accomplished utilizing a homemade roll-mill machine. In this process, 22.5 kg of hardened steel balls (Ø = 25mm) and 1 kg of NiO powder were loaded in the roll-mill container (15 L). The container was evacuated for 10 hours to ensure an absolutely contamination-free condition. Subsequently, the container was charged with N_2_O gas (2 bar) and securely sealed. The roll mill process was conducted at a rotation speed of 115 rpm. for 5 h. The roll-mill was paused for 10 min every one hour to prevent bulk temperature rise. Upon the completion of the reaction, the gas product underwent analysis of online gas chromatography. To verify the absence of any contamination, a blank test was executed by substituting N_2_O with argon. The energy consumption associated with the mechanochemical reaction was quantified by using a digital electrometer (SJPM-C16, SEOJUN) to evaluate the energy efficiency.

**Continuous system using attrition-mill**

Continuous mechanochemical N_2_O decomposition was achieved using a custom-made attrition-mill. The attrition mill container, with a capacity of 1 L, was loaded with 1750 g of ZrO_2_ balls (Ø = 5mm) and 415 g of NiO powder. Prior to milling, N_2_O gas was introduced into the container at a rate of 10 SCCM for a sufficient duration to confirm the removal of any air contamination. The gas flow rate was validated by a mass flow controller (MF-200C, MFC flow) at the gas inlet and a flow meter (G6691A, Agilent) at the gas outlet. Under a continuous gas flow, the mechanochemical N_2_O decomposition was initiated by activating the system at 900 rpm under atmospheric pressure. Following the reaction, the milling system was halted, while the flow of N_2_O gas was maintained. The gas product from the system outlet was subsequently analyzed by an online gas chromatograph. The system temperature was maintained at a constant level of 25 °C using a water chiller.

**Diesel engine exhaust gas treatment using compact mill**

A diesel engine (4 HP, FD170F, Xinweixin) exhaust outlet was connected to a diesel particulate filter (DPF) and a homemade compact mill (width = 60 mm, length = 250 mm). Additional N_2_O gas was introduced into the diesel particulate filter (DPF) system to mix with exhaust gases. The N_2_O gas flow was adjusted by the mass flow controller (MFC). The N_2_O gas flow was adjusted by the mass flow controller (MFC). The mill (volume = 500 mL) was loaded with 200 g of stainless-steel balls (Ø = 5mm) and 250 g of NiO powders. And, a medical grade carbon dioxide (CO_2_) absorber (medical grade, Surgivet) was installed between compact mill and FT-IR gas cell to prevent CO_2_ interference to N_2_O signals. The outlet of compact mill (mechanochemical reduction, MCR, system) was connected with an FT-IR gas cell (162-2250, PIKE), N_2_O (SKZ1050, SKZ) and NO_x_ detectors (Testo 350K, Testo). The exhaust gases were analyzed by in-situ FT-IR spectra, N_2_O and NO_x_ detectors in real time.

**Characterizations**

Commercial Ni_2_O_3_ (99%, US Research Nano) was purchased and used without any post-treatment as a reference sample. Raman spectra were recorded using Alpha300R of WITec. Electron paramagnetic resonance (EPR) spectra were measured with EMXplus (Bruker). Powder x-ray diffraction (PXRD) was obtained using D/MAX2500V from the Rigaku company. The target material was a copper (Cu) target, the Kα ray wavelength was 0.15418 nm. High-resolution transmission electron microscopy (HR-TEM) observations were performed on model JEM-2100F (JEOL). BET adsorption isotherms were obtained using BELSORP-max. Solid-state ultraviolet-visible spectroscopy (UV-Vis) was performed using a Cary 5000 of the Agilent company. Reference material was polytetrafluoroethylene (PTFE). X-ray photoemission spectra (XPS) were obtained on the K-alpha model of Thermo Fisher Scientific Inc., and XPS spectra were calibrated with the C 1s peak at 284.6 eV. Electron energy loss spectroscopy (EELS) was studied by JEM-ARM300F (JEOL) on a Cs corrected Scanning Transmission Electron Microscope (Cs-STEM). Oxygen temperature programmed desorption mass spectrometry (O_2_-TPD-MS) analysis was performed on a BELCAT II (MicrotracBEL). The NiO obtained after ball-milling was analyzed without exposure to O_2_ gas, because enough oxygen gas was already adsorbed on the surface of the sample during the ball-milling process. Following this, we flowed helium gas (30ml min^−1^) at 50 °C for one hour before ramping up the temperature to 900 °C at a rate of 10 °C min^−1^ in a helium gas condition. Optical information of samples was characterized using a ESCALAB 250XI (Thermo Fisher Scientific). Infrared (IR) spectra were measured using a Perkin-Elmer Spectrum 100.

**Theoretical calculation**

The DFT calculations presented in this work were carried out with the Dmol3 software package,^[1]^ which is part of Materials Studio 2017. We utilized the Perdew-Burke-Ernzerhof (PBE) variant of the generalized gradient approximation (GGA) functional to capture the exchange-correlation interactions.^[2,3]^ For addressing the van der Waals forces, the Grimme's DFT-D correction was implemented.^[4]^ The initial spin configuration for Ni was set according to its formal spin state. The computational treatment of the core electrons was based on DFT semi-core pseudopotentials,^[5]^ while the valence electrons were described using a double numerical plus polarization (DNP)^[1]^ basis set with an effective cutoff of 4.4 Å. The slab models all underwent geometry optimization, adhering to convergence thresholds set at 2.0 × 10^−5^ hartree for energy change, 2.0 × 10^−3^ hartree⋅Å^−1^ for force gradient, and 5.0 × 10^−3^ Å for atomic displacement.

In the process of determining the energy barrier for the transition states (TS) of key reaction steps, a linear synchronous transit (LST) maximum was first calculated, which was succeeded by minimizing the energy along directions that were conjugate to the reaction pathway.^[6]^ Subsequently, the approximate TS derived from the LST/optimization process was utilized as a starting point for a quadratic synchronous transit (QST) maximization, which served to pinpoint more precise transition states.

The structure of the model was depicted using a five-layered slab model based on a 2 × 2 × 1 primitive cell of the NiO (200) surface. The three bottom layers of the slab were immobilized throughout the calculations. Owing to the periodicity of the slab model, a 15 Å vacuum gap was added to mitigate any interactions brought about by periodicity. A uniform Monkhorst-Pack k-point grid of 4 × 4 × 1 was selected for sampling the Brillouin zones. Utilizing the computational hydrogen electrode (CHE) model formulated by Nørskov *et al*.,^[7]^ it is possible to compute the Gibbs free energy change (Δ*G*) for various reaction intermediates, which can be calculated by

Δ*G* = Δ*E* + Δ*ZPE* – *T*Δ*S*

where Δ*E* is the adsorption energy difference of intermediates, Δ*ZPE* is the zero-point vibrational energy difference, Δ*S* is the entropy change and *T* denotes the system reaction temperature (298.15 K in this work). Furthermore, for entropy values of specific small molecular gases like N_2_O, N_2_, and O_2_, referencing the CRC Handbook of Chemistry and Physics^[8]^  is considered suitable.

The adsorption energy of reaction intermediates (*E*_ads-*x_) can be calculated by

*E*_ads-*x_ = *E*_*x_ - *E*_*_ - *E*_x_

where *E*_*x_, *E*_*_ and *E*_x_ represent the total energy of the catalyst with and without adsorbates, as well as the corresponding adsorbates in gas phase, respectively.

**Figures:**





**Figure S1.** Evolution of N_2_ to O_2_ ratio during repeated ball milling cycles. When pristine NiO is initially reacted with N_2_O, oxygen from N_2_O is chemically adsorbed onto the NiO surface in the form of O^*^, leading to a stoichiometric reaction. In this initial stage, the N_2_ to O_2_ ratio is significantly high, reaching approximately 7. However, as the reaction cycles progress, the NiO surface becomes increasingly saturated with O^*^. In the sixth cycle, the N_2_ to O_2_ ratio stabilizes near the theoretical value of 2 for N_2_O decomposition, indicating the transition from stoichiometric to catalytic reaction. All experimental data presented in this paper were collected after stable catalytic performance was achieved.





**Figure S2.** Gas chromatography spectrum of the blank test using planetary ball-mill. Spectrum magnified by 350 times corresponding to nitrogen (N_2_), oxygen (O_2_) and N_2_O regions indicated that there were no notable peaks attributable to contaminations.

**

**

**Figure S3.** Comparative mechanochemical performance of different catalysts. The N_2_O decomposition rates of various catalysts were investigated under the same mechanochemical conditions.





**Figure S4.** Mechanochemical and thermochemical N_2_O decomposition performances of Co_3_O_4_, CuO, and Fe_2_O_3_. (a) Co_3_O_4_, (b) CuO, and (c) Fe_2_O_3_. Unlike NiO, these catalysts did not exhibit a significant improvement under mechanochemical conditions. This difference can be explained by several factors. NiO is inherently more active for N_2_O decomposition compared to other catalysts due to its lower activation energy. This lower activation energy is attributed to its favorable properties for the N_2_O dissociative adsorption and oxygen desorption processes.^[9]^ In addition, the influence of activation energy on the reaction rate becomes more critical at the low temperatures typical of mechanochemical systems according to Arrhenius equation. Furthermore, as the mechanochemical N_2_O decomposition progresses, the surface of NiO undergoes gradual transformation into an ultra-oxidized state, enhancing its catalytic activity. In contrast, such transformations are minimal in Co_3_O_4_, CuO, and Fe_2_O_3_ due to their limited initial activity. Therefore, while mechanochemical approach can activate various metal oxides to some extent, the extent of enhancement strongly depends on the intrinsic properties of each material.





**Figure S5.** Comparison of N_2_O conversion between mechanochemical and thermochemical decomposition. The ball-milling process was carried out at 550 rpm for 22min under 2 bar N_2_O. The temperature in the mechanochemical method was controlled by adjusting rotations per minute (r.p.m.) at a fixed milling time of 22 min. The error bar represents the standard deviation obtained from five independent experiments.





**Figure S6.** Gas chromatography analysis of mechanochemical N_2_O decomposition. The spectral range of N_2_O in the boxed area was magnified 900 times. GC results were obtained by analyzing the gas products of the mechanochemical reaction, after the reactor was charged with N_2_O (2 bar) and processed at 550 rpm for 22 minutes. The volume ratio between N_2_ and O_2_ in the gas products approached 2, which corresponds well to the theoretical value expected when N_2_O was decomposed.





**Figure S7.** Studies on thermochemical parameters (catalyst loading, flow rate, contact time, and inhibitor). (a) N_2_O conversion with different total amount of NiO catalyst. Reduced catalyst amount led to a corresponding decrease in N_2_O conversion, confirming that catalyst loading was an important factor in thermochemical N_2_O decomposition. (b) N_2_O conversions with different flow rates and contact times in thermochemical N_2_O decompositions. The impact on flow rate and contact time was investigated by varying the flow rate to 5, 10, and 15 sccm, corresponding to 300, 600, and 900 mL g^−1^ h^−1^ GHSV, respectively. The results showed that, as the flow rate and GHSV increased, the N_2_O conversion decreased. This indicates that flow rate and GHSV are critical factors in thermochemical processes, affecting the contact time between N_2_O and the catalyst, which in turn influences the overall conversion efficiency. (c) N_2_O conversions with and without 10% O_2_ in the feed stream in thermochemical N_2_O decompositions. The N_2_O conversion at around 450 °C decreased from 49.2% to 40.5% with the inclusion of O_2_, indicating that O_2_ acted as an inhibitor in thermochemical N_2_O decomposition processes.





**Figure S8.** Influence of initial N_2_O pressure on N_2_O conversion. The N_2_O conversion was independent of the initial pressure. The error bars represent the standard deviation obtained from five independent experiments.





**Figure S9.** Correlation between N_2_O decomposition and ball size ratio. The effect of ball ratio was investigated by mixing 3 mm and 5 mm balls. Increasing the proportion of 5 mm balls enhanced the collision energy, although it led to a decrease in the number of collisions.





**Figure S10.** Mechanochemical N_2_O decomposition with different total amounts of NiO catalyst. Reactions using 24 g and 1 g of NiO catalysts showed 52.2% and 3.8% N_2_O conversions, respectively.





**Figure S11.** N_2_O decomposition rates of pristine NiO (NiO-r, reference) and post-processed NiO samples (NiO-m, mechanochemical treatment) as a function of ball milling time under N_2_O atmosphere.





**Figure S12.** EPR measurements of pristine (NiO-r, reference) and post-processed NiO samples (NiO-m, mechanochemical treatment) as a function of ball milling time under N_2_O atmosphere.


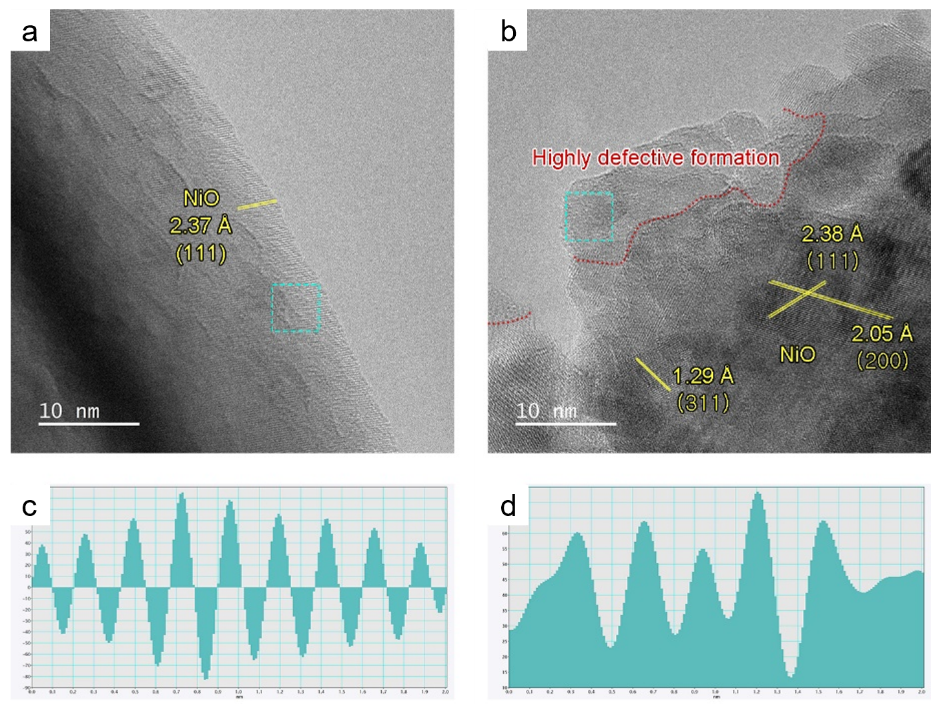


**Figure S13.** TEM analysis. (a) HR-TEM image of NiO-r. The calculated d-spacing from the HR-TEM image of NiO-r corresponds to the lattice spacing of NiO(111) facet. (b) HR-TEM image of NiO-m. The surface regions of NiO-m undergo a transformation into the highly defective state (red line), while the bulk matrix of NiO-m retains its original state. This behavior arises because of the surface-selective transformation nature of the ball-milling process. In addition, the surface, which contains diverse types of defects, is extended by repeated dynamic collisions during the ball-milling process. This phenomenon contributes to the increase in defects. (c,d) Profiles of IFFT for NiO-r and NiO-m were, respectively, acquired from analyses of the dotted blue boxes located near the surfaces of the corresponding samples.

**
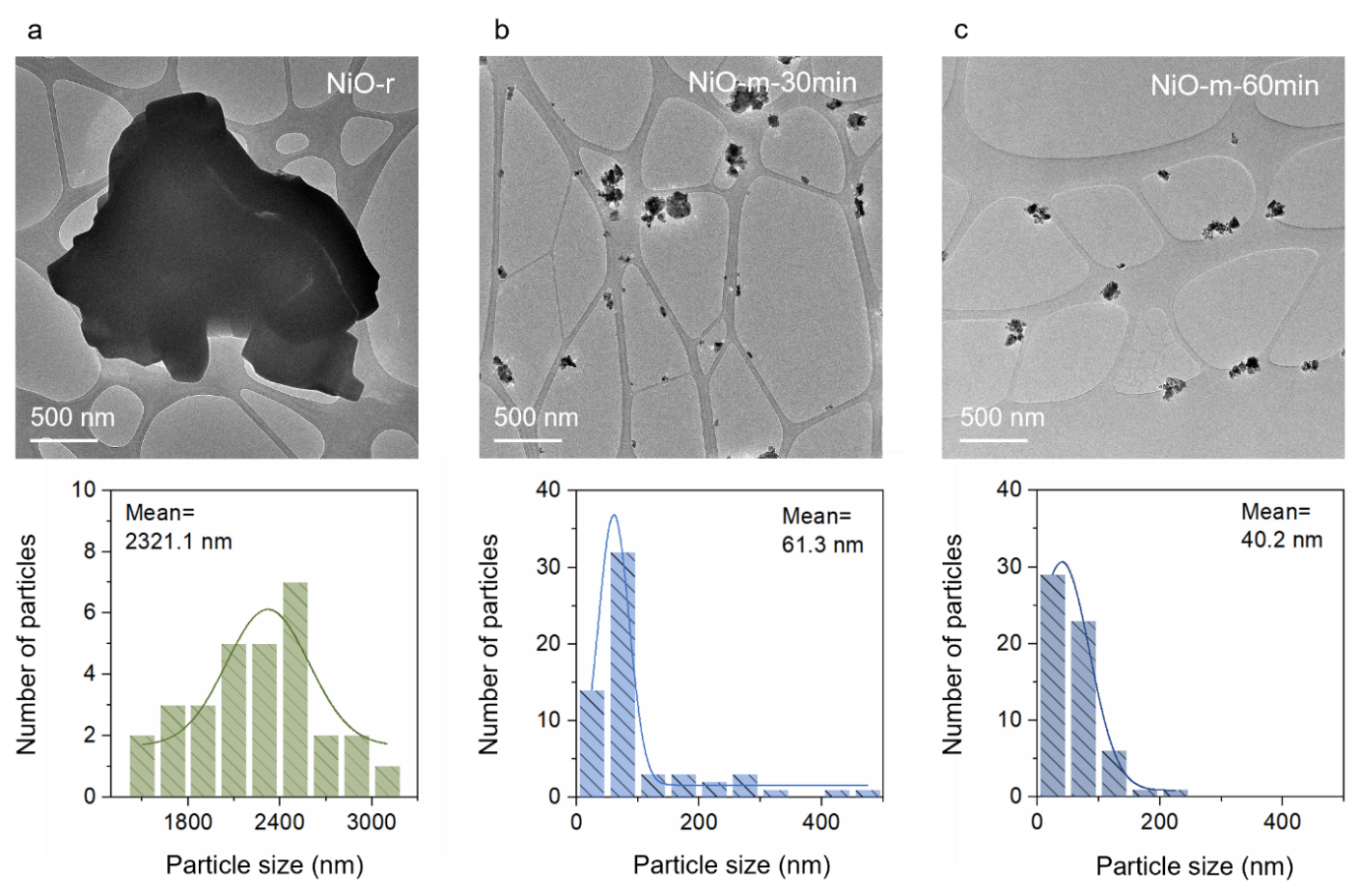
**

**Figure S14.** TEM images and particle size distributions of samples. (a) NiO-r, (b) NiO-m-30min, (c) NiO-m-60. The particle size of NiO decreases rapidly during the initial stages of milling and gradually approaches saturation, reducing from 2321.1 nm to 40.2 nm. The particle size cannot decrease indefinitely with extended ball milling, because it eventually reaches equilibrium between comminution and reconstruction.





**Figure S15.** Validation of defect contributions to enhanced N₂O decomposition. (a) GC analysis of gas products obtained after charging N_2_O (2 bar) into a ball-milling jar containing pristine NiO catalyst, which was then left undisturbed for 10 h. Prior to N_2_O charging, the jar was evacuated for 2 h. A negligible amount of nitrogen (0.1%) was detected, confirming minimal contamination. (b) GC analysis was conducted using the same procedure as in (a), except that the catalyst used was defective NiO. To ensure a high density of defects, defective NiO was prepared by ball-milling pristine NiO under an argon atmosphere at 550 rpm for 5 h. The ball-milling jar was vacuum-sealed and directly charged with N_2_O to prevent air exposure. In contrast to (a), a meaningul amount of nitrogen was detected, indicating that the first dissociative adsorption step of N_2_O was facilitated by the generated defects.





**Figure S16.** UV-vis spectra for quantifying the optical characteristics of samples. Inset: corresponding photographs of samples. The NiO-m sample was diluted with an equal volume of polytetrafluoroethylene (PTFE), because of its too strong absorbance close to 1.





**Figure S17.** UV-vis spectra of NiO-m at various dilution levels. Optical spectra of NiO-m diluted with polytetrafluoroethylene (PTFE) at different levels (0, 2, 5, 10-fold).

**

**

**Figure S18.** Ni L_3_/L_2_ ratios obtained from electron energy loss spectroscopy. The white line of the 3d transition Ni metal was subjected to background subtraction and intensity normalization

**

**

**Figure S19.** Thermodynamic stability of nickel oxides at different oxidation states. The calculated formation energies were obtained using computational methods.^[10-14]^





**Figure S20.** Gibbs free energy of adsorption of N_2_O and desorption free energy of the first N_2_ on NiO with and without defects. Here, the defects primarily consist of vacancies (O-vacancy and Ni-vacancy, abbreviated as O_v_ and Ni_v_, respectively) and single heteroatoms (O and Ni atoms, abbreviated as O_add_ and Ni_add_). Due to the mixed surface nature of NiO (200), with alternating bonding between Ni and O atoms, O_add_ or Ni_add_ have two possible point defect formation sites on the NiO surface. Specifically, the O_add_ (Ni_add_) atom can bond with either Ni or O atoms of the defect-free NiO. Additionally, the linear N_2_O molecule has four probable adsorption configurations on a defect-free NiO surface and twelve probable adsorption configurations on a NiO surface with defects.

Based on careful calculations, we found that the majority of catalysts exhibited stronger adsorption after the introduction of defects, implying better activation for N_2_O. However, Δ*G*_N2_ simultaneously became more positive, which is an unfavorable outcome for the first N_2_ to desorb under ambient conditions from a thermodynamic perspective. According to the Sabatier principle, an ideal scenario would involve a moderate Δ*G*_N2O_ and a more negative Δ*G*_N2_.^[15]^

Fortunately, this was consistent with the aforementioned discussion when N_2_O decomposition occurred on the NiO surface with a Ni_add_ heteroatom bonded to the intrinsic Ni atom. In this case, the Δ*G*_*N2O_ for the terminal O atom of N_2_O bonding with Ni_add_ was -2.58 eV, which is more negative than the terminal N atom bonding with Ni_add_ (-0.93 eV), indicating that N_2_O will preferentially adsorb in the former manner. Simultaneously, Δ*G*_N2_ was -3.03 eV, implying that the first N_2_ can spontaneously dissociate.





**Figure S21.** Initial N_2_O adsorption structure diagram and corresponding energy barrier for the first N_2_ desorption on certain NiO surfaces. To deeply explore N_2_O decomposition, five possible reaction pathways for dissociating the first N_2_ were screened. The O-Ni_add_ (adding Ni to Ni) configuration exhibited the lowest energy barrier (0.91 eV) among the five configurations, and this barrier was significantly reduced after the introduction of the heteroatom Ni, compared to N-Ni (defect-free) (2.52 eV). Therefore, the desorption of the first N_2_ is also feasible under dynamic conditions.

**
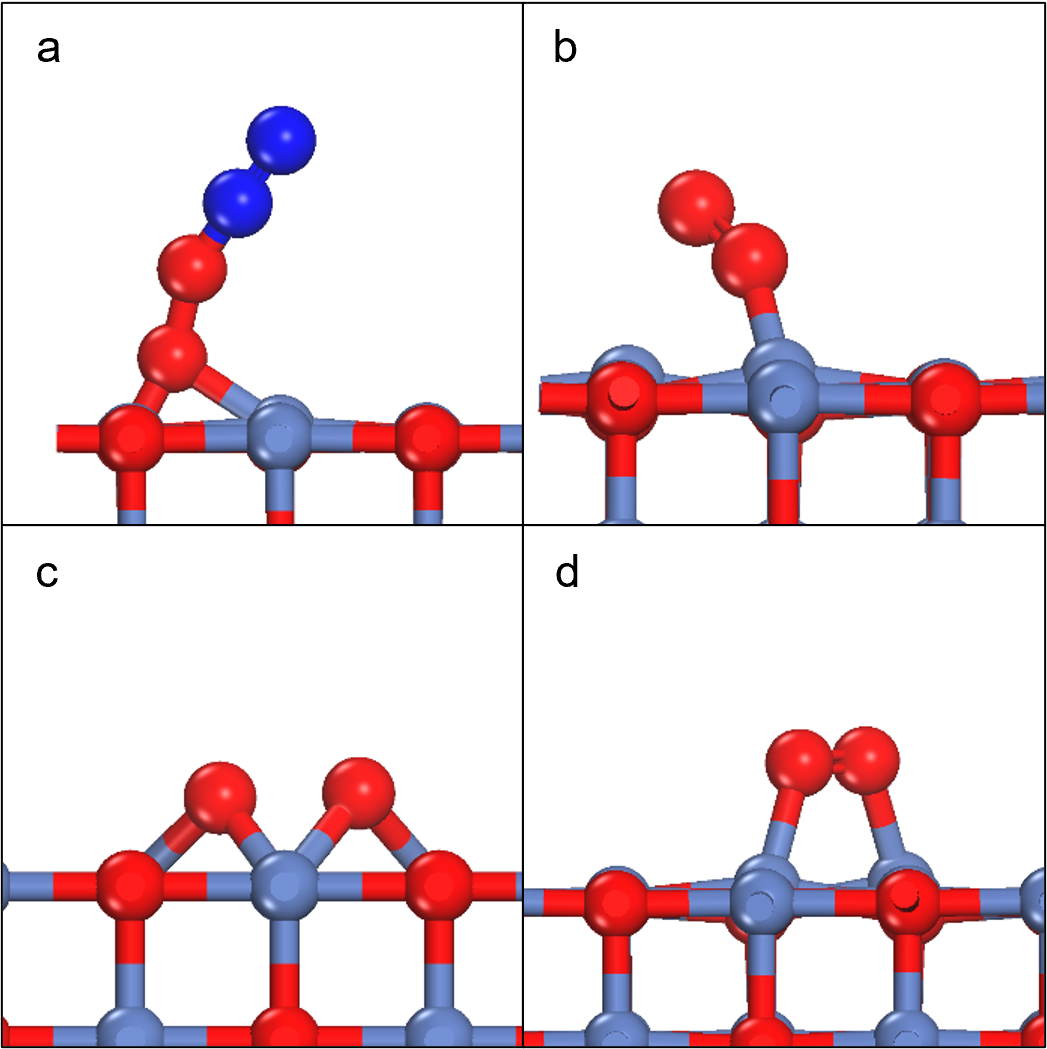
**

**Figure S22.** Schematic representations of structure models for DFT calculation of oxygen desorption step. Structure models of (a) O* and (b) *O_2_ (end-on) for O* + N_2_O → O_2_ + N_2_ pathway. Structure models of (c) O* and (d) *O_2_ (side-on) for O* + O* → O_2_ pathway.





**Figure S23.** Control experiments to evaluate the contribution of O_2_ desorption pathways. To further evaluate the contribution of the O* + O* → O_2_ pathway, we conducted a control experiment. NiO was pre-milled under O_2_ atmosphere for 5 hours to saturate its surface with oxygen species. This sample was then subjected to a short 5 minutes milling in either N_2_O atmosphere (NiO-O_2_-N_2_O) or Ar atmosphere (NiO-O_2_-Ar), these control experiments designed to minimize N_2_O dissociation (step 1) and isolate the effect of the oxygen desorption step (step 2). As shown in Figure Sx, the amount of O_2_ released from NiO-O_2_-Ar was only 0.9 mL, representing the O* + O* → O_2_ pathway. In contrast, the NiO-O_2_-N_2_O sample released 15.1 mL of O_2_, highlighting the additional contribution from the O* + N_2_O → O_2_ + N_2_ pathway. These results collectively support that while the O* + O* → O_2_ mechanism does contribute to active site regeneration to some extent, the O* + N_2_O → O_2_ + N_2_ pathway is dominant under mechanochemical N_2_O decomposition conditions.





**Figure S24.** Control experiments to evaluate rate-determining step (RDS) of mechanochemical N_2_O decomposition. In the NiO-Ar-N_2_O experiment, NiO was pre-milled under Ar for 5 hours to generate a large number of defects, followed by a short 5 minutes milling in N_2_O atmosphere. This condition was designed to isolate step 1 (N_2_O → O* + N_2_). This experiment released 64.4 mL of N_2_ with negligible O_2_, supporting that step 1 dominated under this condition. In the NiO-O_2_-N_2_O experiment, NiO was pre-milled under O_2_ for 5 hours to saturate the surface with oxygen species, followed by the same short milling in N_2_O. This condition was designed to isolate step 2 (O* + N_2_O → O_2_ + N_2_). This experiment released 14.0 mL of N_2_, along with a nearly 1:1 ratio of N_2_ and O_2_, indicating that step 2 was dominant. These results demonstrate that step 2 is the RDS of the overall reaction.


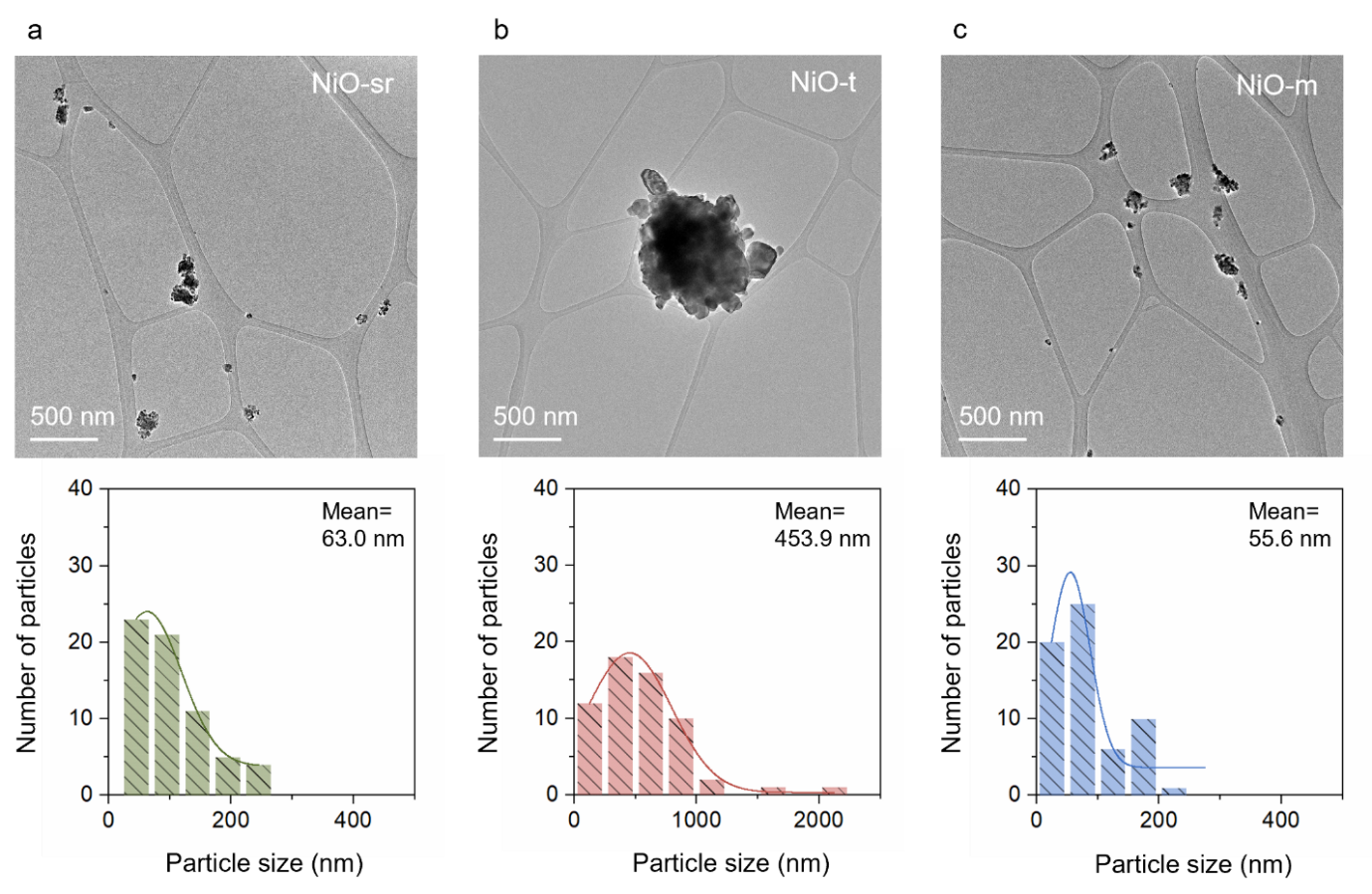


**Figure S25.** TEM images and particle size distributions of samples before and after N_2_O decomposition reactions. (a) Size-reduced NiO (NiO-sr). (b) Thermochemically treated NiO (NiO-t). (c) Mechanochemically treated NiO (NiO-m). NiO-sr was prepared under argon gas atmosphere at the same conditions of NiO-m under N₂O atmosphere. This NiO-sr was used for thermochemical N₂O decomposition reaction, ensuring a fair comparison between mechanochemical and thermochemical methods by minimizing the difference in surface areas. The results demonstrated significant differences between the two samples. After thermochemical treatment, the particle size of NiO-t increased from 63.0 nm to 453.9 nm, which aligns with the theory of Ostwald ripening. This result suggests that, during thermochemical reaction, the NiO-sr catalyst particles undergo agglomeration, leading to reduced active sites and a loss of catalytic activity at high temperatures. In contrast, the particle size of NiO-m remained nearly unchanged compared to NiO-sr, indicating that the ‘dynamic’ mechanochemical process maintains particle size by reaching an equilibrium between comminution and aggregation. This equilibrium prevents significant particle agglomeration and preserves the active surface area during the reaction.





**Figure S26.** BET adsorption isotherms of samples before and after N_2_O decomposition reactions. (a) NiO-sr, (b) NiO-t, (c) NiO-m. BET measurements revealed that NiO-sr, NiO-t, and NiO-m exhibited Type IV isotherms, characteristic of mesoporous.^[16]^ This classification is supported by the following features observed in the isotherms. In high relative pressure region (P/P₀ > 0.8), a sharp increase in adsorption was observed, attributable to pore condensation. In addition, the presence of a hysteresis loop further confirmed the mesoporous structures with capillary condensation within the pores.

**

**

**Figure S27.** Stability tests of the mechanochemical method under various gas mixtures. Stability tests were conducted using gas mixtures resembling flue gases: (a) adipic acid production. (b) nitric acid production, (c) three ways catalysts. The error bars represent the standard deviation obtained from three independent experiments. For each cycle, a gas mixture was introduced at 2 bar and milling was conducted at 550 rpm for 22 minutes using 24 g of NiO catalyst and 450 g of ZrO_2_ balls (1:1 mixture of 3 mm and 5 mm balls in diameter). The stability tests were performed by repeating this process five times.

**
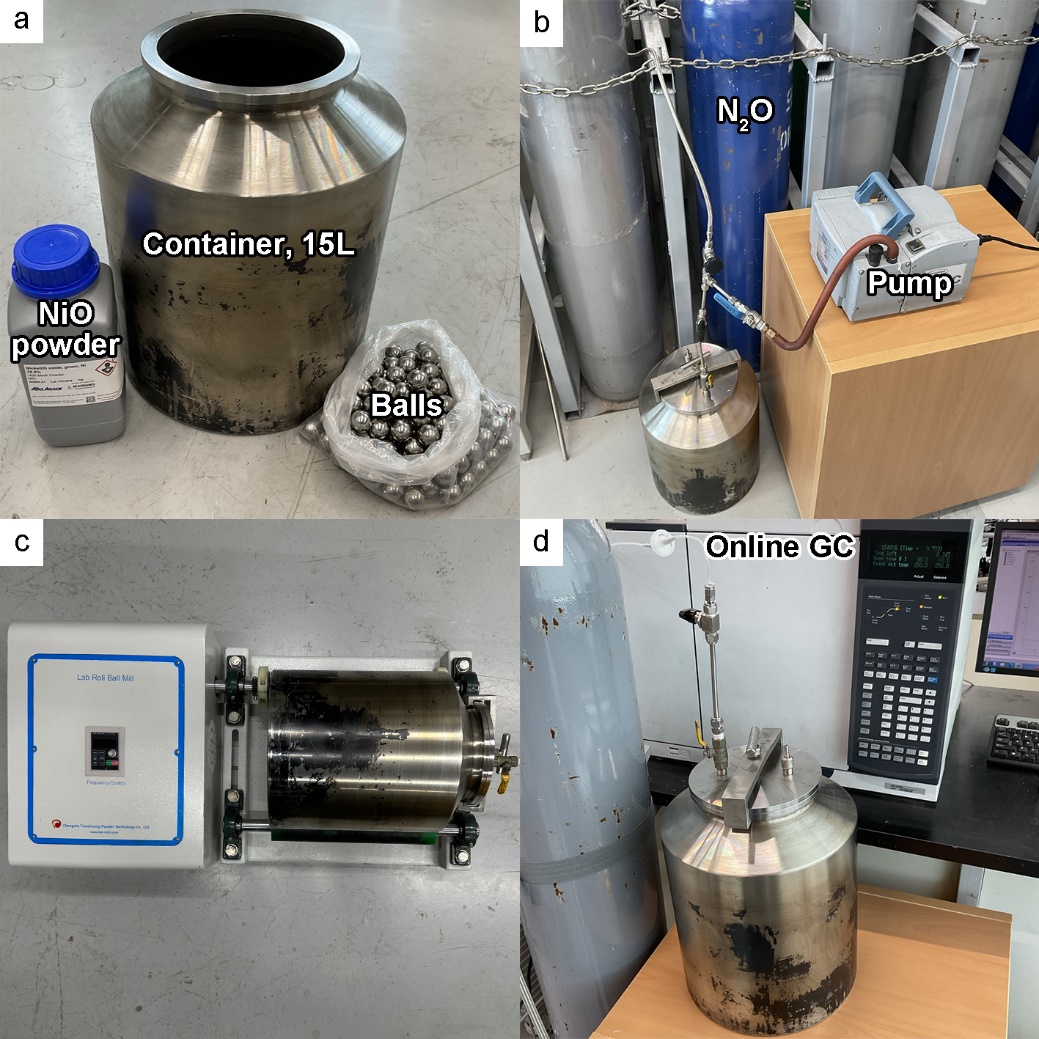
**

**Figure S28.** Roll-mill operating procedure for mechanochemical N_2_O decomposition. (a) Load the hardened steel balls and nickel oxide powder in the milling jar (15L). (b) Evacuate the container interior for 10 h and charge N_2_O gas (2 bar). (c) N_2_O decomposition using roll-mill equipment. (d) Analysis of the gas products using online gas chromatograph.

**

**

**Figure S29.** Gas chromatography spectrum of the blank test using the roll-mill. The spectrum range magnified by 350 times corresponding to nitrogen (N_2_), oxygen (O_2_) and N_2_O indicated that there were no significant peaks attributed to contaminations.


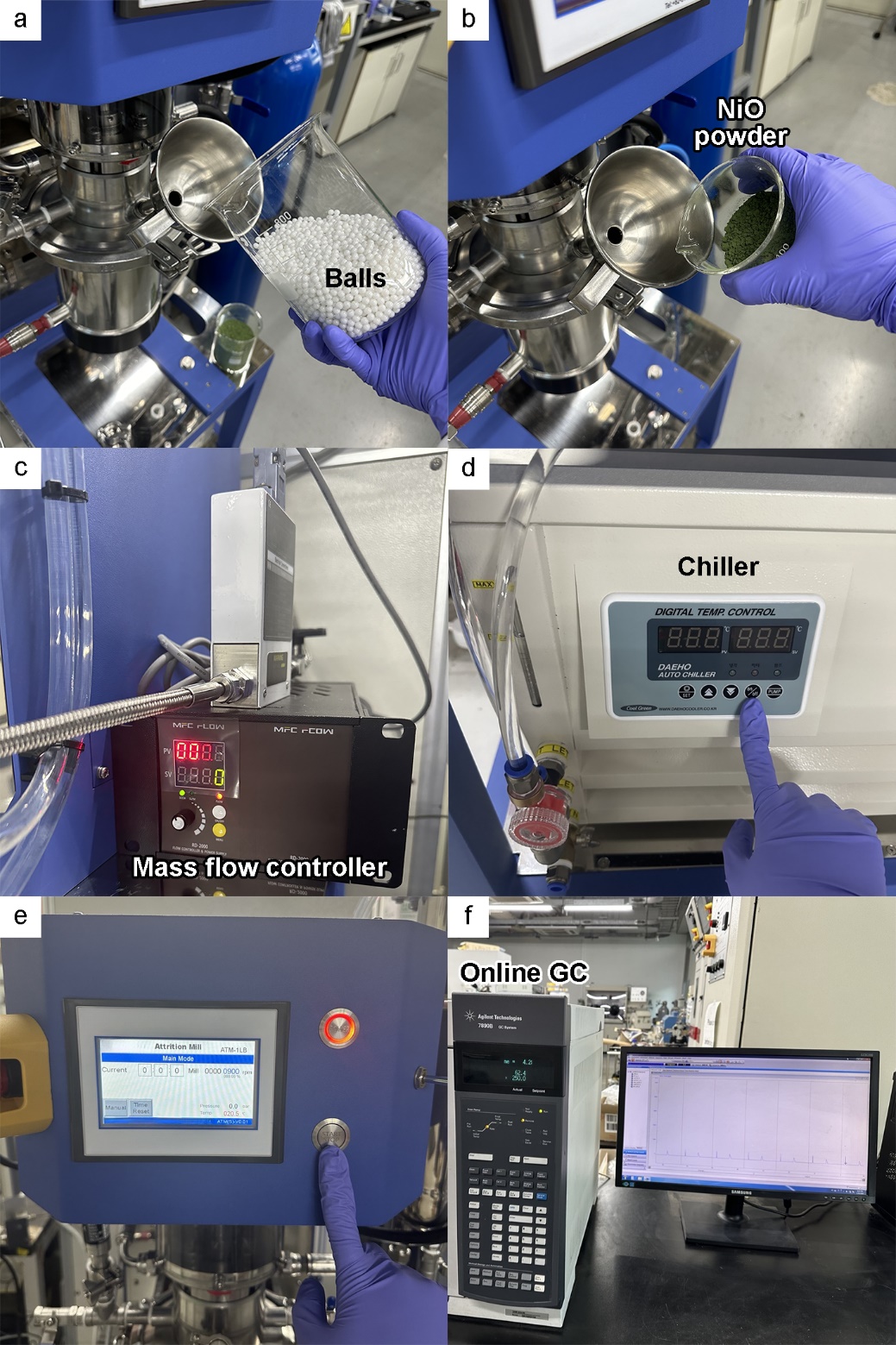


**Figure S30.** Continuous mechanochemical N_2_O decomposition process using home-made attrition mill. (a) Charging ZrO_2_ balls and (b) and NiO powders in the container, the container lid is sealed and the N_2_O gas line is connected to the gas inlet. (c) Controlling the flow rate of N_2_O at 10 SCCM and double checking the value with a flow meter attached to the gas outlet. (d) Turning on the water chiller to maintain system temperature. A circulating coolant maintains a constant system temperature. (e) Starting the milling system at 900 rpm. (f) Sequentially, measuring the gas products with online GC connected to the gas outlet of the milling container in real time.

**
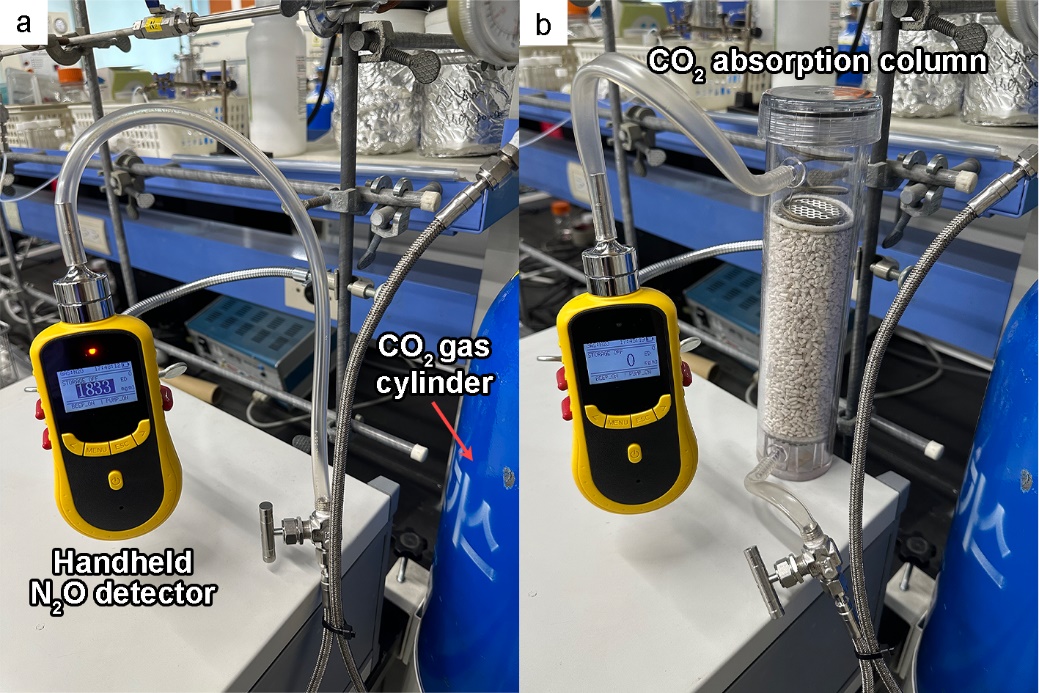
**

**Figure S31.** CO_2_ absorber for exclusion of interference effect to N_2_O signals. (a) CO_2_ flow to handheld N_2_O detector without CO_2_ absorber. Handheld N_2_O detector, which displayed 1833 mg/m^3^, sensed CO_2_ as N_2_O because of interference effect. (b) CO_2_ flow to handheld N_2_O detector with CO_2_ absorber. N_2_O concentration of 0 mg/m^3^ confirmed the successful exclusion of CO_2_ interference effect.

**

**

**Figure S32.** Comparison of N_2_O detection with and without CO_2_ trap. N_2_O gas was passed through the CO_2_ trap, and the result was compared to a control experiment without the trap using FT-IR analysis. There is no significant difference between the two cases. The N_2_O peak area comparison confirms this observation, with values of 0.56 for the result with the CO_2_ trap and 0.58 for the result without the CO_2_ trap. These results indicate that the CO_2_ trap selectively adsorbs CO_2_ without affecting N_2_O concentration.


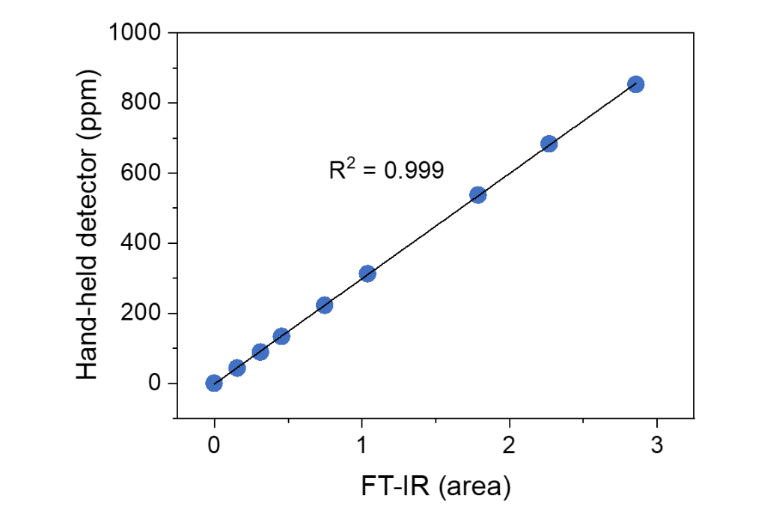


**Figure S33.** Correlation between hand-held N_2_O detector and FT-IR spectroscopy. The reliability of the hand-held N_2_O detector was verified by cross-checking with FT-IR spectroscopy. The N_2_O peak area measured by FT-IR was compared with the N_2_O concentration displayed by the hand-held detector. The comparison revealed high reliability, with an R^2^ value of 0.999, indicating a strong correlation between the two different measurement techniques.

**

**

**Figure S34.** NO_x_ concentration with respect to operation time. The concentration of NO_x_ was measured by NO_x_ detector using a Testo 350K,^[17]^ showing approximately 70% of continuous NO_x_ reduction after mechanochemical activation of NiO for approximately 150 s.

**
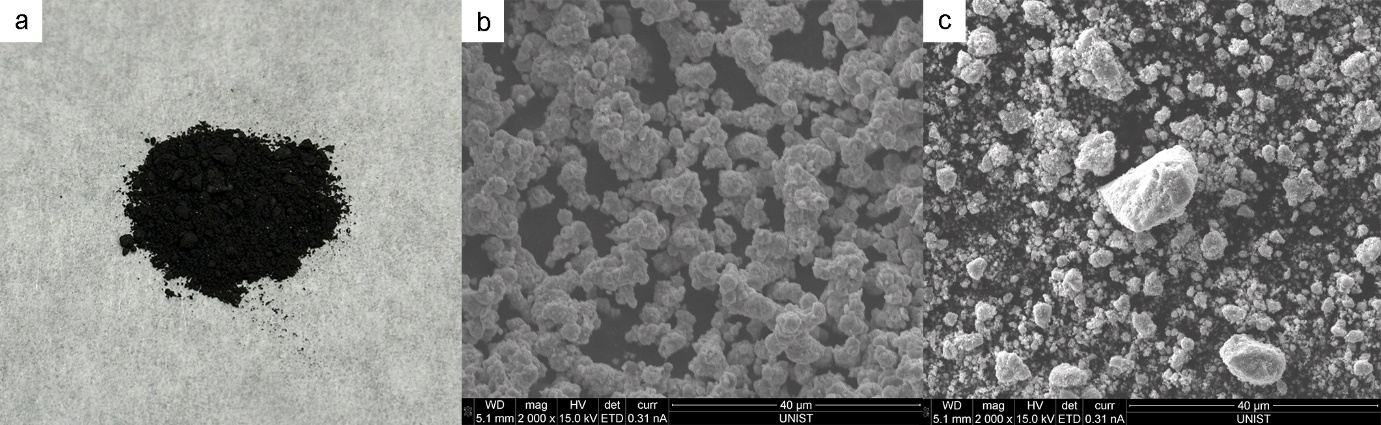
**

**Figure S35.** NiO catalyst recovered after mechanochemical N_2_O decomposition experiment using MCR system. (a) Digital photograph of NiO recovered after MCR treatment. Scanning electron microscope (SEM) images: (b) before, and (c) after MCR treatment.

**
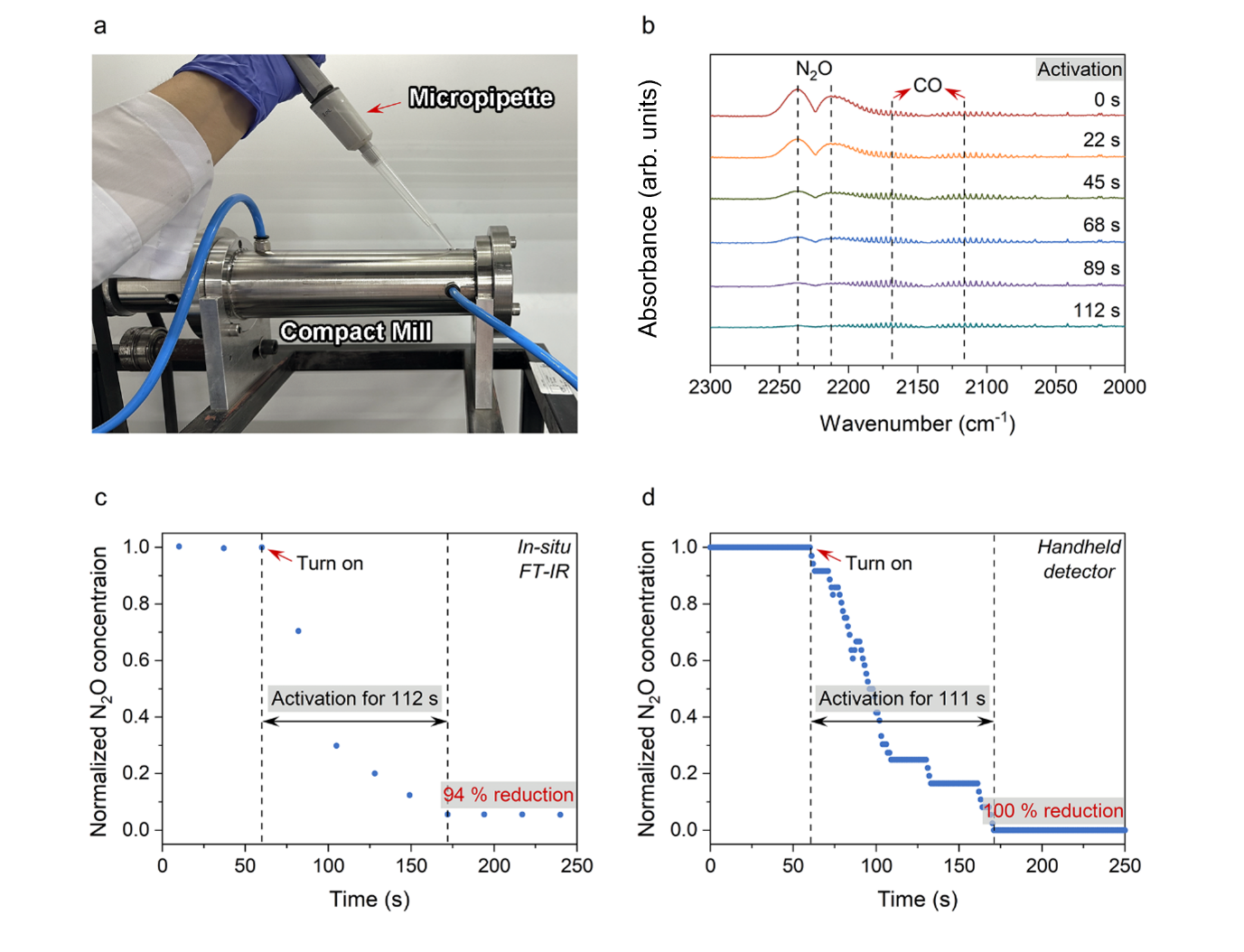
**

**Figure S36.** Condensed water effect associated with the MCR performance. (a) Additional water loading of 5.3 mL into the compact mill with micropipette. Loading amount was calculated by exhaust gas composition of diesel engine.^[18]^

$$Charging water amount \left( mL \right)= \frac{F_{exhaust gas}\times x_{H_{2}O}\times M_{H_{2}O}\times t}{V_{m}\times\rho_{H_{2}O}}$$

where *F* is the gas flow of diesel engine exhaust gas (1 L min^−1^), $x$ is the mole fraction of H_2_O in exhaust gas (0.11), *M* is the molar mass of H_2_O (18 g mol^−1^), *t* is the test time of MCR (60 min), *V_m_* is the gas molar volume (22.4 L mol^−1^), and *ρ* is the density of water (1 g mL^−1^). (b) *In-situ* FT-IR spectra. (c) Corresponding N_2_O concentration response. Under the same experimental condition except water loading, negligible performance degradation compared with control experiment (Fig. 4f-i) was confirmed by *in-situ* FT-IR spectra. Activation time was displayed above corresponding each spectrum. (d) N_2_O concentration response using handheld N_2_O detector.

**Tables:**

**Table S1.** Working catalysts of mechanochemistry and thermochemistry. "Working catalyst" is fundamentally different between mechanochemistry and thermochemistry. In thermochemistry, the entire catalyst is uniformly activated by heat, meaning that all the catalyst can participate in the reaction. Therefore, the total catalyst equals the working catalyst in thermochemical processes. However, in mechanochemistry, the reaction predominantly occurs during the collision process, meaning that only a small portion of the catalyst in the collision region is actively involved in the reaction.^[19,20]^ Thus, the term "working catalyst" in mechanochemistry refers specifically to the fraction of total catalyst that participates in the reaction within these localized collision zones. In our experiments, we observed that the ball-milled catalyst adhered to the surface of the balls due to its high surface energy. Using optical microscopy, we measured the diameter of the collision region to be approximately 0.15 mm.^[21]^ By calculating the ratio of the collision region area to the average surface area of the milling balls, we determined that the working catalyst accounts for approximately 0.08% of the total catalyst used

| Method | Mechanochemistry | Thermochemistry |  |
| --- | --- | --- | --- |
|  |  |  |  |
|  |  |  |  |
| Working catalyst | Localized to collision regions | Entire catalyst |  |
|  |  |  |  |
|  |  |  |  |
| Catalyst utilization | Small fraction of total catalyst | Full utilization of catalyst |  |
|  |  |  |  |
|  |  |  |  |
| Activation source | Mechanical energy from collisions | Thermal energy (heat) |  |
|  |  |  |  |
|  |  |  |  |

**Table S2.** N_2_O decomposition rate based on normalized, same working, same total catalyst amounts. When the N_2_O decomposition rate is measured by the same total catalyst amount, thermochemistry shows a higher reaction rate than mechanochemistry. However, when measured by the same working catalyst amount, mechanochemistry demonstrates a significantly higher reaction rate than thermochemistry. This highlights the unique catalytic activation can be achieved by the mechanochemical process

| Method | Mechanochemical N_2_O decomposition rate (μL g^−1^s^−1^) | Thermochemical N_2_O decomposition rate (μL g^−1^s^−1^) |  |
| --- | --- | --- | --- |
|  |  |  |  |
|  |  |  |  |
|  |  |  |  |
| Normalized catalyst amount | 25481.3 | 81.9 |  |
|  |  |  |  |
|  |  |  |  |
|  |  |  |  |
| Same working catalyst amount (0.02 g) | 25481.3 | 355.1 |  |
|  |  |  |  |
|  |  |  |  |
|  |  |  |  |
| Same total catalyst  amount (1 g) | 35.4 | 81.9 |  |
|  |  |  |  |
|  |  |  |  |
|  |  |  |  |

**Table S3.** Surface oxygen concentration determined by XPS analyses

| Sample | NiO-r | Ni_2_O_3_ | NiO-m |
| --- | --- | --- | --- |
| O concentration^[a]^ (at/at %) | 65.8 | 66.7 | 74.9 |

1. Even though oxygen might adsorb on the surface of the nickel oxide during the transfer of samples for analysis, the observed results still allow us to infer the underlying trends.

**Table S4.** Textural properties of NiO-sr, NiO-t and NiO-m measured by BET. NiO-sr and NiO-m exhibit similar textural properties, including surface area, pore size, and pore volume, whereas NiO-t shows significant degradation in all textural properties except pore size compared to NiO-sr (Figures S25 and S26). In case of pore size increase by thermochemical reaction, it seems the agglomeration of particles makes empty spaces between particles. These results suggest that the thermochemical method leads to catalytic activity decay at high temperatures, while the mechanochemical method maintains stable catalytic activity. This observation is consistent with TEM results

| Samples | NiO-sr | NiO-t | NiO-m |  |
| --- | --- | --- | --- | --- |
|  |  |  |  |  |
|  |  |  |  |  |
| Surface area (m^2^ g^-1^) | 21.423 | 3.481 | 21.286 |  |
|  |  |  |  |  |
|  |  |  |  |  |
| Pore size (nm) | 16.264 | 39.735 | 16.299 |  |
|  |  |  |  |  |
|  |  |  |  |  |
| Pore volume (cm^3^ g^-1^) | 0.087 | 0.035 | 0.086 |  |
|  |  |  |  |  |
|  |  |  |  |  |

**Table S5.** Mixture gas compositions

| Chemical process | Gas composition (%)^[a]^ | | | |
| --- | --- | --- | --- | --- |
|  | N_2_O | NO_2_ | O_2_ | CO |
| Adipic acid production | 50 | 0.7 | 4 | 0.03 |
| Nitric acid production | 0.35 | 0.18 | 2.5 | - |
| Three ways catalysts  (TWC) | 0.1 | 0.1 | 0.05 | 0.2 |

1. Mixture gases were formulated based on the gas compositions found in real flue gases from industries.^[22]^

**Table S6.** Comparison between thermochemistry and mechanochemistry

| Method | Thermochemistry | Mechanochemistry | Mechanochemistry |  |
| --- | --- | --- | --- | --- |
|  |  |  |  |  |
| Process classification | Continuous | Batch | Continuous |  |
|  |  |  |  |  |
| Reaction condition | 1 bar, 445 °C | 2 bar, 34 °C | 1 bar, 25 °C |  |
|  |  |  |  |  |
|  |  |  |  |  |
| Decomposed N_2_O (mmol) | 13.2 | 78.6 | 26.2 |  |
|  |  |  |  |  |
| Consumed energy^[a]^ (kWh) | 0.276 | 0.270 | 0.132 |  |
|  |  |  |  |  |
| **Energy efficiency (mmol/kWh)** | **47.710** | **291.215** | **198.150** |  |
|  |  |  |  |  |

1. Consumed energies for reactions were measured by digital electrometer.

**Table S7.** Economic assessment for mechanochemical N_2_O decomposition

| **Description** | **Mechano** |
| --- | --- |
| **0. Decomposition rate (mmolN_2_O/y)** | **632,908.43** |
| **1. Capital cost ($/y)** | **3,093.18** |
| Mill | 1,670.01 |
| Container | 541.62 |
| Milling Ball | 360.00 |
| Catalyst (NiO) | 6.02 |
| Supplement | 515.53 |
| **2. Operating cost ($/y)** | **1,625.21** |
| Electricity | 280.49 |
| Labor | 734.26 |
| Maintenance | 406.97 |
| Other costs | 203.49 |
| **3. Total cost ($/y)** | **4,718.39** |

**Table S8.** Economic assessment for thermochemical N_2_O decomposition

| **Description** | **Thermo** |
| --- | --- |
| **0. Decomposition rate (mmolN_2_O/y)** | **105,988.37** |
| **1. Capital cost ($/y)** | **4,349.54** |
| Fixed-bed reactor | 3,610.82 |
| Quartz tube | 13.54 |
| Catalyst (NiO) | 0.25 |
| Supplement | 724.92 |
| **2. Operating cost ($/y)** | **2,179.58** |
| Electricity | 286.72 |
| Labor | 1,032.50 |
| Maintenance | 573.57 |
| Other costs | 286.79 |
| **3. Total cost ($/y)** | **6,529.12** |

**Video:**

**Video S1.** Exhaust gas treatment from a diesel engine using a homemade compact mill. The structure of the system consists of a diesel engine, diesel particulate filter (DPF), homemade compact mill (MCR), CO_2_ adsorption column, FT-IR gas cell, and gas detectors.

**References**

[1] B. Delley, "From molecules to solids with the DMol^3^ approach" *J. Chem. Phys.* **2000**, 113, 7756-7764.

[2] S. Grimme, "Semiempirical GGA‐type density functional constructed with a long‐range dispersion correction" *J. Comput. Chem.* **2006**, 27, 1787-1799.

[3] J. P. Perdew, K. Burke, M. Ernzerhof, "Generalized gradient approximation made simple" *Phys. Rev. Lett.* **1996**, 77, 3865.

[4] S. Grimme, J. Antony, S. Ehrlich, H. Krieg, "A consistent and accurate ab initio parametrization of density functional dispersion correction (DFT-D) for the 94 elements H-Pu" *J. Chem. Phys.* **2010**, 132, 154104.

[5] B. Delley, "Hardness conserving semilocal pseudopotentials" *Phys. Rev. B* **2002**, 66, 155125.

[6] T. A. Halgren, W. N. Lipscomb, "The synchronous-transit method for determining reaction pathways and locating molecular transition states" *Chem. Phys. Lett.* **1977**, 49, 225-232.

[7] J. K. Nørskov, J. Rossmeisl, A. Logadottir, L. Lindqvist, J. R. Kitchin, T. Bligaard, H. Jonsson, "Origin of the overpotential for oxygen reduction at a fuel-cell cathode" *J. Phys. Chem. B* **2004**, 108, 17886-17892.

[8] W. M. Haynes, *CRC Handbook of Chemistry and Physics, 96th Edition*, CRC Press, **2016**.

[9] F. Zhao, D. Wang, X. Li, Y. Yin, C. Wang, L. Qiu, J. Yu, H. Chang, "Enhancement of Cs on Co_3_O_4_ for N_2_O Catalytic Decomposition: N_2_O Activation and O_2_ Desorption" *Ind. Eng. Chem. Res.* **2022**, 61, 13854-13862.

[10] M. Aykol, S. S. Dwaraknath, W. H. Sun, K. A. Persson, "Thermodynamic limit for synthesis of metastable inorganic materials" *Sci Adv* **2018**, 4, eaaq0148.

[11] A. Jain, G. Hautier, S. P. Ong, C. J. Moore, C. C. Fischer, K. A. Persson, G. Ceder, "Formation enthalpies by mixing GGA and GGA + U calculations" *Phys. Rev. B* **2011**, 84, 045115.

[12] R. Kingsbury, A. S. Gupta, C. J. Bartel, J. M. Munro, S. Dwaraknath, M. Horton, K. A. Persson, "Performance comparison of r^2^ SCAN and SCAN metaGGA density functionals for solid materials via an automated, high-throughput computational workflow" *Phys. Rev. Mater.* **2022**, 6, 013801.

[13] R. S. Kingsbury, A. S. Rosen, A. S. Gupta, J. M. Munro, S. P. Ong, A. Jain, S. Dwaraknath, M. K. Horton, K. A. Persson, "A flexible and scalable scheme for mixing computed formation energies from different levels of theory" *Npj Comput. Mater.* **2022**, 8, 195.

[14] A. Wang, R. Kingsbury, M. McDermott, M. Horton, A. Jain, S. P. Ong, S. Dwaraknath, K. A. Persson, "A framework for quantifying uncertainty in DFT energy corrections" *Sci. Rep.* **2021**, 11, 15496.

[15] E. J. Karlsen, L. G. M. Pettersson, "N_2_O Decomposition over BaO:  Including Effects of Coverage" *J. Phys. Chem. B* **2002**, 106, 5719-5721.

[16] E. S. Tarleton, *Progress in Filtration and Separation*, Academic Press, **2015**.

[17] UIPA (Ulsan Information Industry Promotion Agency) Equipment Portal, Retrieved May. 20, 2024, from http://ermh.uipa.or.kr/).

[18] W. Addy Majewski, Khair, Magdi K., *Diesel Emissions and Their Control*, SAE Inernational, **2006**, 122.

[19] A. W. Tricker, K. L. Hebisch, M. Buchmann, Y. H. Liu, M. Rose, E. Stavitski, A. J. Medford, M. C. Hatzell, C. Sievers, "Mechanocatalytic Ammonia Synthesis over TiN in Transient Microenvironments" *ACS Energy Lett.* **2020**, 5, 3362-3367.

[20] A. W. Tricker, G. Samaras, K. L. Hebisch, M. J. Realff, C. Sievers, "Hot spot generation, reactivity, and decay in mechanochemical reactors" *Chem. Eng. J.* **2020**, 382.

[21] G.-F. Han, P. Zhang, P. Scholzen, H.-J. Noh, M. Yang, D. H. Kweon, J.-P. Jeon, Y. H. Kim, S.-W. Kim, S.-P. Han, A. S. Andreev, G. Lang, K. Ihm, F. Li, J.-B. d'Espinose de Lacaillerie, J.-B. Baek, "Extreme enhancement of carbon hydrogasification via mechanochemistry" *Angew. Chem. Int. Ed.* **2022**, 61, e202117851.

[22] F. Kapteijn, J. Rodriguez-Mirasol, J. A. Moulijn, "Heterogeneous catalytic decomposition of nitrous oxide" *Appl. Catal. B.* **1996**, 9, 25-64.
